# The integrated stress response engages a cell-autonomous, ligand-independent, DR5-driven apoptosis switch

**DOI:** 10.1038/s41419-025-07403-8

**Published:** 2025-02-15

**Authors:** Francesca Zappa, Nerea L. Muniozguren, Julia E. Conrad, Diego Acosta-Alvear

**Affiliations:** 1 Department of Cellular, Molecular, and Developmental Biology, University of California, Santa Barbara, USA; 2 Altos Labs Bay Area Institute of Science, Altos Labs, Inc., Redwood City, USA; 3 Present Address: Altos Labs Bay Area Institute of Science, Altos Labs, Inc., Redwood City, USA

**Keywords:** Cell biology, Cell death

## Abstract

The integrated stress response (ISR) is a fundamental signaling network that leverages the cell’s biosynthetic capacity against different stresses to restore homeostasis. However, when homeostasis is unattainable, the ISR switches to drive cell death and eliminate irreparably damaged cells. Previous work has shown that persistent activity of the ISR kinase PERK during unyielding endoplasmic reticulum (ER) stress induces apoptosis downstream of death receptor 5 (DR5) [1]. ER stress provides activating signals that engage the ectodomain (ED) of DR5 to drive its unconventional activation in the Golgi apparatus [1, 2]. Here, using chemical genetics to uncouple stress sensing from ISR activation, we found that DR5 signaling from the Golgi apparatus is integral to the ISR and not specific to ER stress. Furthermore, we show that DR5 activation can be driven solely by increased expression and does not require its ED. These findings indicate that a general ISR kill switch eliminates irreversibly injured cells.

## Introduction

The integrated stress response (ISR) is an intracellular signaling network governed by four sensor kinases—GCN2, HRI, PKR, and PERK—that are activated by specific stresses, including amino acid shortages and ribosome collisions (GCN2), heme deficit, oxidative and mitochondrial stress (HRI), viral and endogenous double-stranded RNAs (PKR), and loss of ER proteostasis (PERK) [3]. These kinases phosphorylate a single serine in the alpha subunit of the eukaryotic initiation factor 2 (eIF2α), leading to a cell-wide protein synthesis shutdown. Some mRNAs harboring upstream open reading frames (uORFs) escape this regulatory control and are selectively translated upon eIF2α phosphorylation. These mRNAs include those encoding the transcription factors ATF4 and CHOP, as well as GADD34, which encodes a regulatory subunit of protein phosphatase 1 that dephosphorylates eIF2α and establishes a negative feedback loop that terminates ISR signaling and establishes the stress memory [4–9].

The ISR reprograms the translatome and transcriptome to meet the cell’s demands and restore homeostasis. However, when restoring homeostasis is impossible during overwhelming, persistent stress, the ISR switches to drive apoptosis to eliminate irreparably damaged cells. During ER stress, opposing signals between PERK and the ER stress sensor kinase/RNase IRE1—one of the three governors of the Unfolded Protein Response (UPR)—dictate adaptive or terminal outcomes [1, 10]. CHOP, downstream of PERK, induces death receptor 5 (DR5), a protein belonging to the Tumor Necrosis Factor (TNF) superfamily of plasma membrane-localized death receptors, which includes DR4, TNF, and Fas. In the early adaptive phase of the UPR, IRE1 cleaves DR5 mRNA to degrade it. Dampening pro-survival IRE1 signals by the phosphatase RPAP2 downstream of PERK during persistent ER stress switches the balance in favor of pro-apoptotic signaling by the PERK-CHOP axis, leading to DR5 activation [10]. In this mechanism, DR5 accumulates in the ER-Golgi intermediate compartment (ERGIC) and signals unconventionally from within the cell without needing its cognate TNF-related apoptosis-inducing ligand (TRAIL). It has been suggested that during ER stress, unfolded proteins accumulate and bind as activating ligands to the ectodomain (ED) of DR5, and disulfide bond rearrangements in DR5’s ED contribute to its activation by oligomerization [1, 2, 11].

These observations suggest that persistent ER stress invokes a local and private cell elimination program. However, eIF2α phosphorylation and CHOP induction are common ISR outputs, which suggests that a general ISR cell-elimination mode governed by DR5 may exist. To test this hypothesis, we exposed cells to multiple ISR-inducers and, in parallel, deployed a stress-input agnostic approach in which we activated the ISR in the absence of stress and analyzed DR5 signaling. Together, our findings substantiate that the terminal ISR operates through a universal, stress-input-agnostic, DR5-dependent kill switch.

## Results

### Different ISR-inducing stresses converge on DR5 activation

**Fig. 1:**
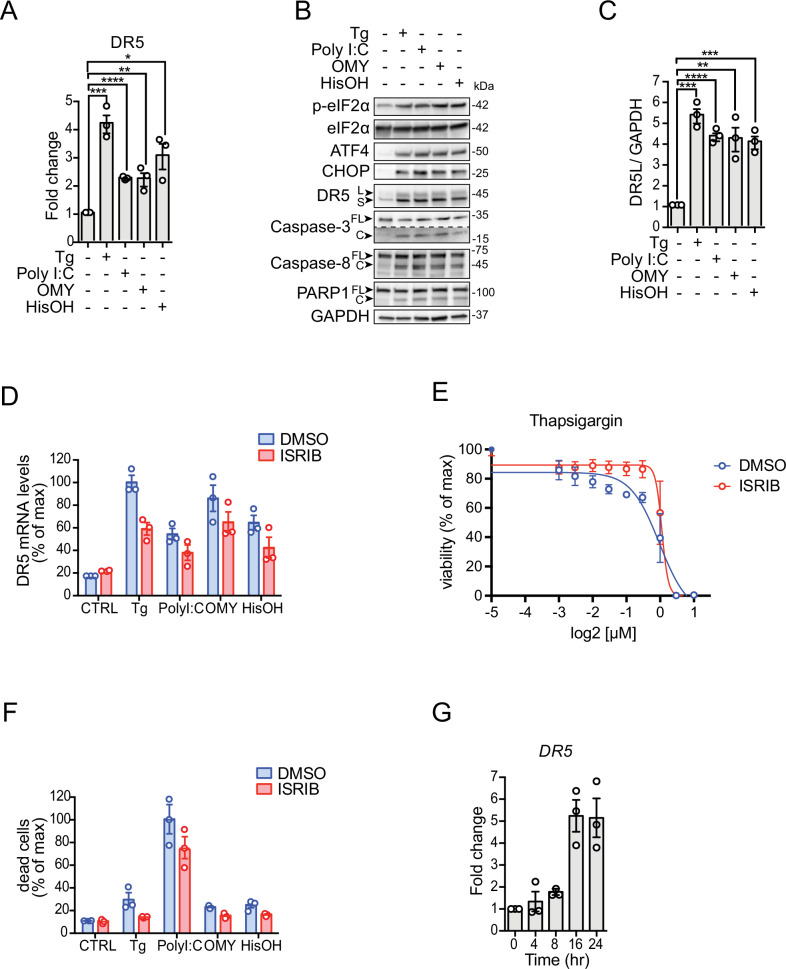
Multiple ISR inputs induce DR5-driven cell death. **A** Quantitative real-time PCR analysis of DR5 mRNA levels in H4 cells after activation of different branches of the ISR. Thapsigargin (Tg) 300 nM, poly I:C 250 ng/ml, oligomycin (OMY) 3 µM, L-histidinol (HisOH) 5 mM (mean and SEM, *N* = 3, ****P < 0.0001, ***P < 0.001, **P < 0.01, *P < 0.05 unpaired Student’s t-test, non-parametric). **B** Western blot showing upregulation of DR5, cleavage of caspase-8, caspase-3, and PARP1, and induction of canonical ISR markers (p-eIF2α, ATF4, and CHOP) in H4 cells upon treatment with different ISR stressors for 18 h. GAPDH: loading control. **C** Densitometry quantification of the Western blot data for DR5 long isoforms (mean and SEM, *N* = 3, ****P < 0.0001, ***P < 0.001, **P < 0.01 unpaired Student’s t-test). **D** qRT-PCR analysis of DR5 mRNA levels after pre-treating H4 cells with the ISR inhibitor ISRIB (mean and SEM, *N* = 3, ****P < 0.0001 Two-way ANOVA). **E** Viability curve of cells treated with different concentrations of Thapsigargin and co-treated with 1 µM ISRIB. EC_70_ Tg = 0.4 µM, EC_70_ Tg+ISRIB = 0.9 µM. **F** Quantification of cell viability analysis using flow cytometry after staining with propidium iodide. The plot shows the induction of cell death in H4 cells upon treatment with classical pharmacological ISR activators and the restoration of cell viability upon ISR inhibition using ISRIB. Data are expressed as a percentage of the maximum value (mean and SEM, *N* = 3, ****P < 0.0001 Two-way ANOVA). **G** qRT-PCR analysis of DR5 mRNA levels in H4 cells expressing eIF2α ^S51D^ (mean and SEM, *N* = 3, ***P < 0.001 One-way ANOVA).

To investigate whether all ISR sensor kinases converge on DR5 expression, we treated H4 neuroglioma cells with various ISR inducers. We chose a neural cell line because a dysregulated ISR has been observed in numerous neuropathologies [12]. To induce ER stress and activate PERK, we treated cells with the ER calcium reuptake inhibitor thapsigargin [13, 14]. To induce dsRNA stress and activate PKR, we transfected cells with the dsRNA mimetic polyinosinic-polycytidylic acid (poly I:C) [15]. To induce mitochondrial stress and activate HRI, we treated cells with the ATP synthase inhibitor oligomycin [16]. Finally, to mimic nutritional deficit and activate GCN2, we treated cells with L-histidinol, a histidine analog alcohol that prevents histidyl-tRNA charging [17]. We chose drug concentrations and exposure times that are typically used to induce stress in cell culture (indicated in the figure legends and in Materials and methods). We next measured DR5 mRNA levels by qRT-PCR after exposing cells to these different ISR inducers for 18 h, which we reasoned would be sufficient to initiate a terminal response based on previous observations [1] (Fig. 1A). These analyses revealed an approximately 4-fold upregulation of the DR5 mRNA in response to ER stress elicited by thapsigargin (Fig. 1A), which is consistent with the upregulation of DR5 mRNA observed in colon cancer cells subjected to persistent ER stress [18]. Poly I:C, oligomycin, and L-histidinol also elevated the levels of the DR5 mRNA, albeit less potently (approximately 2-fold, poly I:C and oligomycin; 3-fold, L-histidinol). We did not detect the mRNA encoding the death receptor DR4, another TRAIL receptor [19], in cells treated with any of the ISR inducers (Fig. S1A), suggesting that DR5 upregulation is ISR-specific.

The increase in DR5 mRNA elicited by ISR inducers we used was mirrored at the protein level (Fig. 1B, C). As observed for the DR5 mRNA, thapsigargin treatment led to the largest effect at the protein level compared to the other ISR inducers (Fig. 1A–C). TRAIL binding to DR5’s ED promotes DR5 trimerization and recruitment of the adaptor protein FADD and procaspase-8 on the cytosolic leaflet of the membrane to nucleate the Death-Inducing Signaling Complex (DISC), which processes procaspase-8 into active caspase-8 [19]. The upregulation of DR5 was accompanied by the processing of procaspase-8, activation of caspase-3, and cleavage of the canonical apoptosis marker PARP1 (Fig. 1B), all of which are consistent with DR5’s pro-apoptotic activity. These changes tracked with canonical activation of the ISR, as evidenced by self-phosphorylation of PERK, PKR, and GCN2, phosphorylation of eIF2α, and induction of ATF4 and CHOP (Figs. 1B, S1B). We could not detect HRI auto-phosphorylation in cells treated with oligomycin since no commercial phospho-HRI antibodies are available, and the detection of phosphorylated HRI by electrophoretic mobility shift proved unreliable.

Given that CHOP induces DR5 [1, 18], our results suggest that the cell death decision is relayed to terminal effectors by eIF2α phosphorylation. If this is the case, inhibition of the ISR should suppress, at least in part, DR5 accumulation and apoptosis. To test this notion, we co-treated cells with the different ISR inducers and the small molecule ISR inhibitor ISRIB, which renders cells insensitive to the effects of eIF2α phosphorylation [20]. ISRIB modestly yet consistently inhibited the upregulation of DR5 mRNA measured by qRT-PCR (Fig. 1D), partially restored cell viability, measured by ATP levels (Figs. 1E, S1C), reduced cell death, measured by propidium iodide (PI) staining (Fig. 1F), and shifted the dose-response curve of thapsigargin (Figs. 1E, S1C; EC_70_ Tg = 0.4 µM, EC_70_ Tg + ISRIB = 0.9 µM) and the more potent oxidative stressor and HRI activator sodium arsenite (SA) (Fig. S1C, EC_70_ SA = 3.0 µM, EC_70_ SA + ISRIB = 20.1 µM). These observations indicate that cell death signals can be bypassed to some extent in ISRIB-treated cells and substantiate the notion that the cell death decision is relayed, at least in part, by the ISR core to terminal effectors.

We reasoned that the incomplete protection by ISRIB we observed in the experiments above stems from the pleiotropic effects of the ISR inducers we employed, which all are potent poisons. To rule out potential confounding effects, we employed a genetics-based approach in which we force-expressed eIF2α^S51D^, a phosphomimetic point mutant of eIF2α, under the control of a tetracycline-regulatable promoter. eIF2α^S51D^ expression led to a robust time-dependent accumulation of DR5 mRNA, starting at 8 h after induction and reaching an approximate 5.5-fold saturation level at 16 h (Fig. 1G), which corresponds with the levels of DR5 we observed in thapsigargin treated cells (Fig. 1A). This time frame is consistent with the expression of eIF2α^S51D^ and consequent ISR activity as measured by immunoblot and qRT-PCR of ISR markers (Fig. S1D–F). Together, these results indicate that DR5 is induced by the different ISR kinases downstream of phosphorylated eIF2α.

### Stress-free activation of the ISR induces DR5 and apoptosis

Our observations that DR5 is induced by activation of any of the ISR kinases beg the question of whether a general molecular circuit controls the terminal ISR. To dissect the molecular determinants exclusive to the terminal ISR and avoid the pleiotropic effects of stress-inducing agents, we employed a validated chemical-genetics approach consisting of an engineered ISR sensor kinase, FKBP-PKR, which can be activated with a small molecule ligand to actuate canonical ISR signaling independently of stress [21]. FKBP-PKR-driven ISR actuation is fast, reversible, and engages GADD34 (compare Fig. S1D–F to Fig. 3 in [21]). By contrast, the forced expression of eIF2α^S51D^ is not reversible by GADD34 and requires transcription and translation, resulting in a lag time. Thus, FKBP-PKR allows a rapid, stress-free activation of a “pure” ISR [21].

**Fig. 2:**
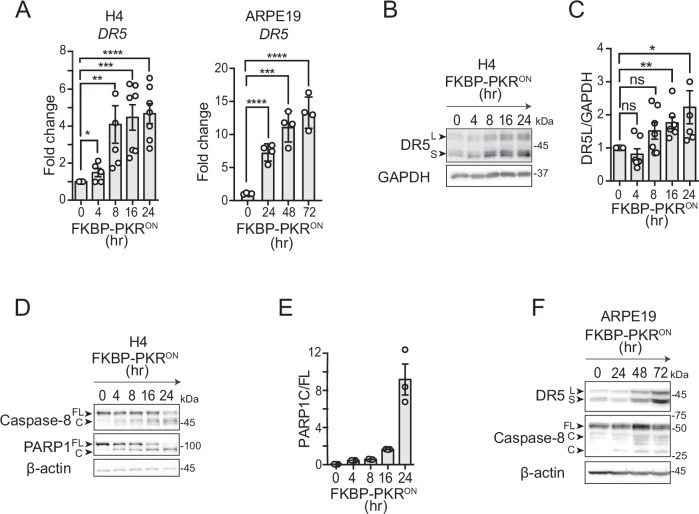
Stress-free activation of the ISR induces DR5 and caspase-8 cleavage. **A** qRT-PCR analysis of DR5 mRNA levels after activation of FKBP-PKR in H4 and ARPE19 cells (mean and SEM, *N* = 4, ****P < 0.0001, ***P < 0.001, **P < 0.01, *P < 0.05 unpaired Student’s t-test). **B** Western blot showing upregulation of DR5 isoforms after activation of FKBP-PKR. GAPDH: loading control. **C** Densitometry quantification of the long DR5 isoform (mean and SEM, *N* = 5, **P < 0.01, *P < 0.05, ns= not significant, unpaired Student’s t-test). **D** Western blot showing caspase-8 and PARP1 cleavage after FKBP-PKR activation. GAPDH: loading control. **E** Densitometry quantification of PARP1 cleavage upon FKBP-PKR activation at the indicated time points (Ratio, mean and SEM, *N* = 3). **F** Western blot showing upregulation of DR5 isoforms and cleavage of caspase-8 after activation of FKBP-PKR in ARPE19 cells. β-actin: loading control.

FKBP-PKR activation in H4 cells led to a time-dependent accumulation of DR5 mRNA, with a greater than 4-fold induction at later time points and peak levels at 16 h after FKBP-PKR activation (Fig. 2A). These observations are consistent with our results in cells treated with thapsigargin and in cells expressing eIF2α^S51D^ (Fig. 1F). The rise in DR5 mRNA levels was mirrored by a time-dependent accumulation of DR5 protein after FKBP-PKR activation (Fig. 2B, C), which once again aligned with results we obtained using ISR-inducing drugs. These results were not restricted to H4 cells, as DR5 mRNA and protein also accumulated in response to FKBP-PKR activation in non-cancerous retinal cells, ARPE19 and RPE1 (Figs. 2A, F and S2B, C), expressing similar levels of FKBP-PKR as those observed in H4 cells (Fig. S2A). These increases in DR5 mRNA and protein levels were accompanied by canonical apoptosis markers, including procaspase-8 processing and cleavage of PARP1 upon FKBP-PKR activation (Figs. 2D–F, S2C). Notably, DR5 expression and apoptosis induction were delayed in ARPE19 and RPE1 cells when compared to H4 cells (Fig. 2A; and compare Figs. 2B, F and S2B), which could be indicative of the enhanced sensitivity of cancer cells towards DR5-induced cell death [22, 23]. Together, these results demonstrate that DR5 can be induced in a stress-input agnostic manner to initiate cell-autonomous apoptosis downstream of the ISR.

### Cell death downstream of the ISR requires DR5

**Fig. 3:**
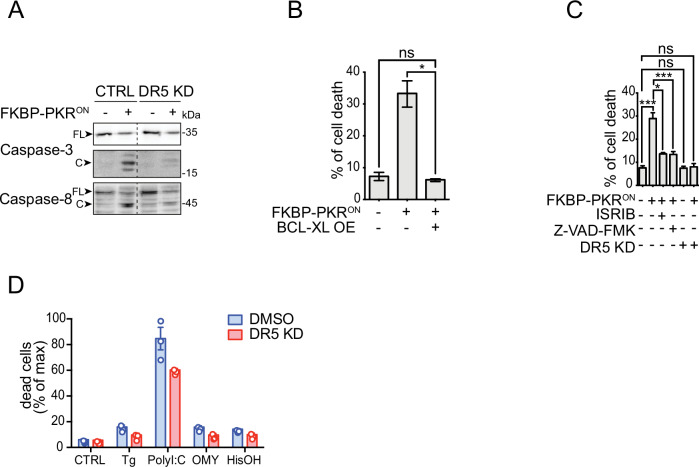
Apoptosis downstream of the ISR requires DR5 and caspase activity. **A** Western blot showing a lack of caspase-8 and caspase-3 activation upon DR5 knockdown in H4 FKBP-PKR activated cells. **B** Flow cytometry quantification of cell death after propidium iodide staining of H4 cells in which we activated FKBP-PKR and overexpressing BCL-XL (mean and SEM, *N* = 3, *P < 0.05, unpaired Student’s t-test). **C** Flow cytometry quantification of cell death after propidium iodide staining in H4 cells treated with the ISR inhibitor ISRIB, the pan-caspase inhibitor Z-VAD-FMK, and upon genetic depletion of DR5 by CRISPRi (mean and SEM, *N* = 3, ***P < 0.001, *P < 0.05, ns= not significant, unpaired Student’s t-test). **D** Flow cytometry quantification of cell death after propidium iodide staining in H4 DR5 CRISPRi cells treated with ISR pharmacological activators (mean and SEM, *N* = 3, ****P < 0.0001 One-way ANOVA).

To test the dependence of the ISR cell death program on DR5, we knocked down DR5 using CRISPR interference (CRISPRi) in H4 cells in cells expressing FKBP-PKR ( > 80% DR5 knockdown efficiency; Fig. S3A–C) and monitored apoptosis upon FKBP-PKR activation. In these experiments, CRISPRi-mediated depletion of DR5 resulted in a substantial decrease in the activation of caspase-8 and caspase-3 (Fig. 3A, note the reduction in the levels of the cleaved form of each caspase, compare lanes 2 and 4), substantiating the notion that DR5 is a primary determinant of ISR-induced apoptosis. To confirm DISC involvement, we depleted FADD using RNA interference. Acute depletion of FADD with approximate 60% efficiency did not prevent caspase-8 processing (Fig. S3D, E), and accordingly, it failed to rescue cell viability (data not shown), indicating that the remaining FADD is sufficient to enforce apoptosis. However, we cannot exclude that other adaptor proteins, for example, TRADD, may link DR5 to caspase processing to drive the apoptosis switch [24].

Apoptosis is controlled by extrinsic (death-receptor-dependent) and intrinsic (mitochondria-permeabilization-dependent) interconnected signaling pathways that converge on the activation of executioner caspases. The pro-apoptotic protein BID, cleaved by caspase-8, bridges the extrinsic and intrinsic pathways [25]. The active, truncated form of BID, tBID, promotes mitochondrial membrane permeabilization and cytochrome c release [37]. To test whether the terminal ISR engages the intrinsic pathway, we stably overexpressed the pro-survival protein BCL-XL, which inhibits mitochondrial membrane permeabilization [26] in cells expressing FKBP-PKR. Forced expression of BCL-XL completely blocked cell death elicited by FKBP-PKR activation as measured by PI staining (Fig. 3B), indicating that cell death signals in the terminal ISR are channeled to the intrinsic apoptosis pathway.

Expectedly, and attesting to ISR involvement, treatment of cells in which we activated FKBP-PKR with ISRIB restored cell viability almost completely, as did treatment with the pan-caspase inhibitor Z-VAD-FMK (~28% cell death down to 12% Fig. 3C compare column 1 to columns 3 and 4, respectively), corroborating ISR and caspase involvement. Notably, the genetic depletion of DR5 by CRISPRi fully restored cell viability in cells in which we activated FKBP-PKR to levels that mirrored those of the untreated controls (approximately 28% cell death down to 8%, Fig. 3C, compare columns 1 and 6). Moreover, the depletion of DR5 alone had no effects on cell viability (Fig. 3C, compare columns 1 and 5). Notably, the knockdown of DR5 modestly yet consistently restored cell viability in H4 cells treated with pharmacological ISR inducers (Fig. 3D), further substantiating the notion that DR5 is required to induce apoptosis in the terminal ISR. We surmise that the effect of DR5 knockdown shown in Fig. 3D was only observed at modest levels because of the pleiotropic effects associated with the potent toxicity of the ISR inducers used.

### Stress-free activation of the ISR leads to intracellular activation of DR5

As mentioned above, during persistent ER stress, DR5 activates intracellularly independently of its ligand TRAIL [1, 2]. Two non-mutually exclusive models for DR5 activation upon unmitigated ER stress have been proposed: unfolded proteins accumulating in the secretory pathway acting as DR5 activating ligands [2] and rearrangement of disulfide bonds in DR5’s ED [11]. Building on these findings, we investigated whether DR5 accumulates intracellularly and signals similarly when the ISR is induced synthetically.

**Fig. 4:**
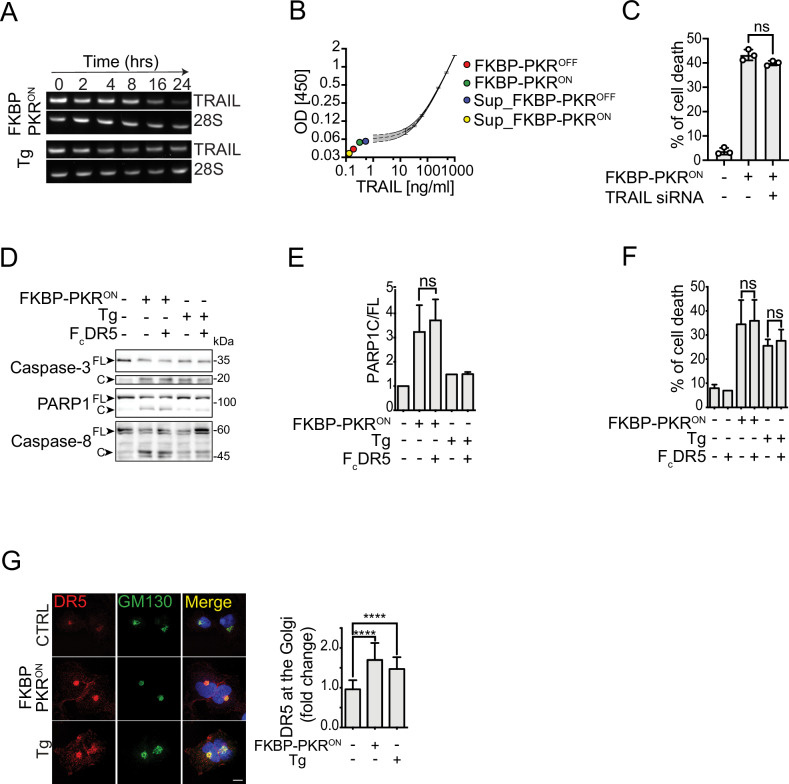
Stress-free activation of the ISR leads to intracellular activation of DR5. **A** RT- PCR analysis showing TRAIL mRNA levels in FKBP-PKR activated and thapsigargin treated cells at different times. 28S: loading control. **B** ELISA showing undetectable levels of TRAIL in cells and media of FKBP-PKR activated cells. **C** Flow cytometry quantification of cell death by propidium iodide staining of TRAIL-depleted H4 FKBP-PKR cells. **D** Western blot showing that blocking plasma membrane DR5 with FcDR5 does not impede the activation of caspase-3, caspase-8, and PARP1 cleavage in H4 cells in which we activated FKBP-PKR. **E** Densitometry quantification of PARP1 cleavage upon activation of FKBP-PKR and co-treatment with FcDR5 (mean and SEM, *N* = 3, ns = not significant, unpaired Student’s t-test). **F** Flow cytometry quantification of cell death after propidium iodide staining of H4 cells treated with FcDR5 or thapsigargin (Tg, positive control). **G** Representative immunofluorescence images showing that DR5 co-localizes with the *cis*-Golgi apparatus marker GM130 upon induction of the ISR with natural stress inputs or in stress-free conditions after activation of H4 FKBP-PKR. Right panel: quantification of the extent of localization of DR5 in the *cis*-Golgi apparatus in immunofluorescence analyses (mean and SEM, *N* = 3, *n* > 1000, ****P < 0.001, *P < 0.05, unpaired Student’s t-test).

To rule out a potential involvement of TRAIL, we measured the levels of TRAIL mRNA by RT-PCR in FKBP-PKR-activated and thapsigargin-treated cells and found that TRAIL mRNA levels decrease upon FKBP-PKR activation (Fig. 4A). It is likely that this drop in TRAIL mRNA levels, which is mirrored by a drop in 28S rRNA levels, results from cell death, as the timing is consistent with caspase activity (Fig. 2D, E). Next, we measured intra and extracellular TRAIL protein levels in FKBP-PKR activated cells and found no detectable secreted TRAIL (Fig. 4B). Finally, we knocked down TRAIL in FKBP-PKR cells and found that TRAIL depletion did not rescue cell viability upon FKBP-PKR activation (Figs. 4C, S4A). To corroborate that H4 cells are susceptible to killing by TRAIL, we tested the ability of recombinant TRAIL to induce cell death in H4 FKBP-PKR cells. Expectedly, treatment with recombinant TRAIL in cells where FKBP-PKR is not active led to a dose-dependent loss in cell viability, indicating engagement of plasma-membrane death receptors (Fig. S4A, blue trace). FKBP-PKR activation by itself led to a drop of ~80% in cell viability at 24 h (Fig. S4B; first point, no TRAIL), which was enhanced when recombinant TRAIL was introduced at concentrations higher than 4.7 ng/ml, which far exceeded what has been observed in human plasma [27] (Fig. S4B, red trace). Collectively, these findings suggest that TRAIL is not necessary for the activation of DR5 in the terminal ISR.

To test whether plasma membrane-localized DR5 signals apoptosis in cells undergoing a terminal ISR—a condition to which H4 cells are susceptible (Fig. 4D)—we exposed cells in which we activated FKBP-PKR to a DR5-neutralizing Fc antibody fragment (FcDR5). Treatment with FcDR5 did not prevent the activation of caspases-3 and -8, or cleavage or PARP1 (Fig. 4D, E), nor did it block cell death in response to FKBP-PKR activation (Fig. 4F), indicating that plasma membrane DR5 is not required for transducing death signals upon activation of a terminal ISR. Next, we examined the subcellular localization of DR5 upon induction of a stress-free ISR. Activation of FKBP-PKR cells led to an accumulation of DR5 in the *cis*-Golgi apparatus, as evidenced by immunofluorescence analyses (Fig. 4G). Strikingly, the intracellular localization of DR5 to the *cis*-Golgi apparatus elicited by stress-free activation of the ISR was virtually indistinguishable from that caused by ER stress-inducing agents (Fig. 4G and [1, 2]).

Together, the observations that (i) TRAIL is not induced, (ii) plasma-membrane DR5 is not required for enforcing the ISR kill switch, and (iii) accumulation of DR5 in the *cis*-Golgi apparatus all occur upon synthetic ISR activation suggest that the terminal ISR engages an intrinsic, generalized, unconventional, cell-autonomous apoptosis mechanism. Moreover, these results indicate that the ISR kill switch is hard-wired and does not require the molecular damage induced by bona fide ISR-activating stresses.

### DR5 activation does not require its ED

**Fig. 5:**
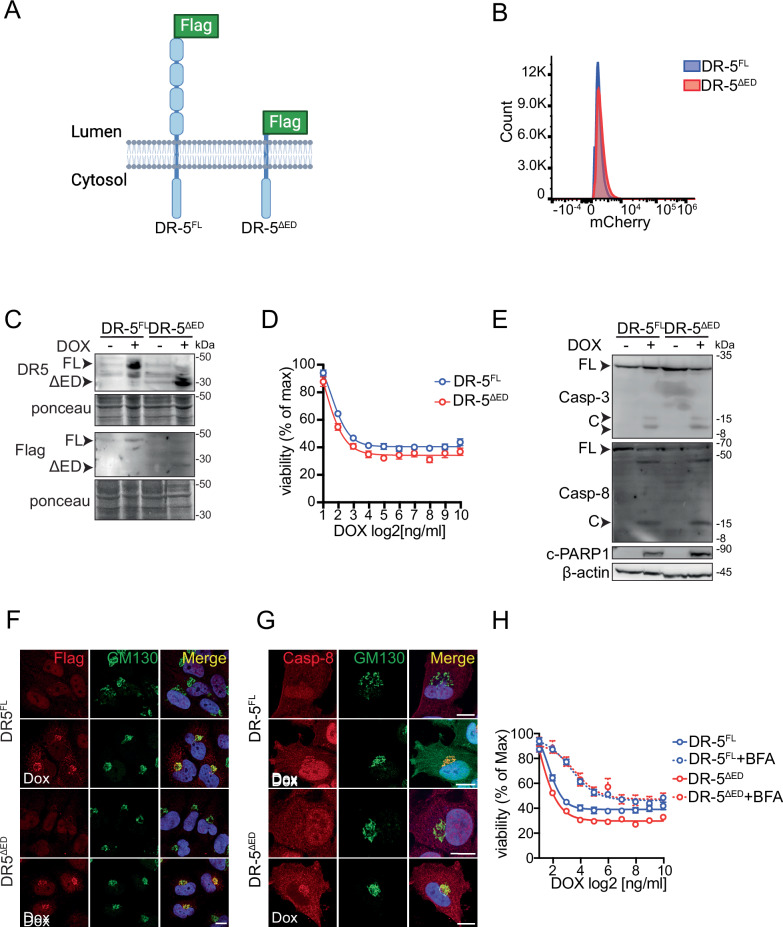
Overexpression of DR5’s intracellular domain is sufficient to drive signaling. **A** Schematic of the two different DR5 protein versions used in this study. DR5^FL^: Full-length DR5; DR5^ΔED^: Truncated form of DR5 lacking the ED. Image created with BioRender.com. **B** Flow cytometry analysis showing the expression of a mCherry tracer encoded in the expression vectors used for DR5 variant overexpression in H4 cells. **C** Western blot showing expression levels of transgenic DR5 proteins. Ponceau: loading control. **D** Viability curves of cells overexpressing transgenic DR5 proteins and exposed to different concentrations of doxycycline (Dox) for 24 h. **E** Western blot showing caspase-8, Caspase-3, and PARP1 cleavage upon expression of DR5 variants (after 24 h). β-actin: loading control. **F** Representative immunofluorescence images showing that DR5 proteins co-localize with the *cis*-Golgi apparatus marker GM130 after 24 h of doxycycline treatment. **G** Representative immunofluorescence images showing that caspase-8 co-localizes with the *cis*-Golgi apparatus marker GM130 after 24 h of doxycycline treatment. **H** Viability curves of cells overexpressing DR5 variants and co-treated with brefeldin-A (BFA, 0.1 μg/ml).

The results above indicate that ISR activation—synthetic or driven by ISR-inducers—increases an intracellular pool of DR5 that signals independently of TRAIL to elicit cell-autonomous apoptosis. Therefore, it is possible that high levels of DR5 and intracellular molecular crowding trigger mass-action-driven signaling, which would not require cognate ligands (i.e., TRAIL or unfolded proteins) or rearrangement of disulfide bonds. To test this hypothesis, we generated cell lines expressing epitope-tagged full-length DR5 and a mutant version lacking the ED under the control of a tetracycline-inducible promoter to titrate their expression. We co-expressed mCherry from the same construct using a constitutive promoter to select cell populations with similar expression levels using fluorescence-activated cell sorting (FACS) and corroborated DR5 expression levels by immunoblot (Figs. 5A–C, S5A). Overexpression of either form of DR5 led to a significant and equivalent, dose-dependent, decrease in cell viability (Fig. 5D). Moreover, both proteins activated the DR5 downstream molecular cascade with similar amplitude, indicated by comparable levels of cleaved caspase-8, caspase-3, and PARP1 (Fig. 5E). Overexpression of neither DR5 transgenic protein was sufficient to induce the UPR, measured by qRT-PCR of the ISR- and UPR-induced genes (*BiP, DNAJB9, CHOP,* and *GADD34*), and splicing of the XBP1 mRNA, a signature of UPR activation (Fig. S5B, C), indicating that the phenotype we observed is unlikely due to ER stress induced by forced expression of these DR5 variants.

Strikingly, both DR5 transgenic proteins accumulated in the *cis*-Golgi apparatus upon induction and co-localized with caspase-8 (Fig. 5F, G). Moreover, collapsing the Golgi apparatus onto the ER with brefeldin A in cells expressing these DR5 variants partially protected them from cell death (Figs. 5H, S5D), indicating that signaling-competent DR5 pools localize to the Golgi apparatus. Together, these results suggest that increasing DR5 levels—by ISR induction or otherwise—is sufficient to assemble the DISC in-situ at the Golgi apparatus membrane to stimulate an intracellular, DR5-dependent cell-autonomous apoptosis program. These findings indicate that DR5 expression alone is enough to engage a cell-autonomous ISR kill switch that does not require intracellular ligands.

## Discussion

Using orthogonal approaches, we demonstrate that the terminal ISR appoints a cell-autonomous apoptosis mechanism that relies on intracellular activation of DR5 without a need for ED-activating signals. We base this conclusion on multiple lines of evidence. First, cell-lethal pharmacologic ISR-inducers upregulated DR5 mRNA and protein (Fig. 1A, B) and actuated apoptosis downstream of DR5 (Fig. 1B), as did the stress-free activation of the ISR using a synthetic biology approach (Fig. 2A–E). Second, the ISR inhibitor ISRIB reversed the upregulation of DR5 and subsequent cell death triggered by the ISR (Figs. 1D, F, 3C), indicating that cell death signals pass through the ISR core, which relies on phosphorylation of eIF2α. Third, cells lacking DR5 were less susceptible to cell death triggered by pharmacologic ISR inducers (Fig. 3D) as well as by a stress-free ISR (Fig. 3C). Fourth, DR5 accumulated in the *cis*-Golgi apparatus in response to thapsigargin and upon stress-free ISR activation (Fig. 4G), and blocking plasma-membrane DR5 with a neutralizing antibody had no effect on cell viability (Fig. 4D–F), indicating intracellular DR5 activation during the terminal ISR. Fifth, the overexpression of an ED-deficient DR5 mutant was sufficient to induce cell death with the same magnitude as full-length DR5 and following the same intracellular activation model (Fig. 5D, E).

For cell health to ensue, the ISR must accurately interpret information about stress states and actuate accordingly to control homeostatic or terminal outputs. On the one hand, tailored homeostatic outcomes are likely executed through ISR kinase signal codes, which could result from additional ISR kinase substrates and interactors and accessibility to different pools of eIF2α. On the other hand, considering all ISR sensor kinases pass signals through the core relay eIF2α, the terminal ISR is likely to employ an off-the-shelf mechanism downstream of eIF2α that funnels information about an irreparable critical state to a common executor, DR5. Our data support this model, indicating that the terminal ISR operates through a kill switch consisting of the pro-apoptotic signaling pathway phospho-eIF2α→CHOP→DR5 [1, 18, 28–30].

Our observation that overexpression of BCL-XL completely prevented cell death caused by the activation of the synthetic ISR suggests the preferential or selective activation of the intrinsic apoptotic pathway within the ISR kill switch. However, cells devoid of Bak, Bax, and caspase-9 can still activate apoptosis in response to unrelenting ER stress [31], suggesting functional redundancies exist to eliminate cells that can no longer reinstate homeostasis, such as combinatorial control and additional pro-cell death roles of stress sensors and transducers. For instance, the ER stress sensors IRE1 and ATF6 may contribute to driving cell death by enlisting autophagy [32, 33].

Our findings that DR5 can be activated by overexpression and in the absence of ISR-inducing stresses suggest that the generalized ISR kill switch does need to match stress inputs to DR5 activation, but rather, that surpassing a threshold during unrelenting stress, which could be ISR signal duration or intensity, is sufficient to engage it. DR5 activation mechanisms have been proposed for unresolved ER stress [1, 2, 10], and while we cannot formally exclude the possibility that any ISR-activating input—including our synthetic ISR activation approach—induces some mild accumulation of unfolded proteins or changes in redox status that may modulate DR5 activity, it is unlikely they do so to a level that is comparable to bona fide ER stresses. Likewise, we cannot rule out the possibility that some assembly of the DISC occurs at the plasma membrane to fine-tune the kill switch.

Signaling fine-tuning considerations notwithstanding, our results argue in favor of DR5 crowding in the *cis*-Golgi apparatus as the principal component of the ISR kill switch, which aligns with previous observations that DR5 overexpression triggers apoptosis [34] and that DR5’s transmembrane domains are sufficient to drive trimerization required for signaling [35]. Considering that the bulk of DR5 protein accumulates in the *cis*-Golgi apparatus upon ISR induction and DR5 overexpression (Figs. 4G, 5F and [1]), it is possible that yet-to-be-discovered mechanisms that retain DR5 in the secretory pathway are integral to part of the ISR kill switch. This is an intriguing possibility that warrants further investigation. Regardless, the identification of a general mechanism controlling cell death during prolonged ISR signaling advances our understanding of how the ISR operates to maintain the health of tissues: customized homeostatic solutions or a one-size-fits-all terminal response.

## Materials and methods

### Cell line engineering, cell culture, genetic knockdown, transduction, and drug treatments

H4 cells stably expressing FKBP-PKR and the CRISPRi machinery have been described previously [21]. H4, ARPE19, and RPE1 were obtained from the American Type Culture Collection (ATCC). The DR5 gene was depleted using CRISPRi as previously described [36]. Briefly, CRISPRi cells were transduced with VSV-G pseudotyped lentiviruses harboring three small guide RNAs (5’-GGGCAAGACGCACCAGTCGT-3’; 5’-GAGAGATGGGTCCCCGGGTT-3’; 5’-GAAAGTAGATCGGGCATCGT-3’). The sgRNA sequences were obtained from the human genome-scale CRISPRi library developed by the laboratory of Jonathan Weissman (MIT, Whitehead Institute). Small interfering RNAs for TRAIL knockdown were commercially obtained and transfected using the manufacturer’s recommendations (Sigma-Aldrich). Additional cell lines stably expressing FKBP-PKR were generated using lentiviruses encoding FKBP-PKR and GFP separated by a P2A peptide. Cells were selected for similar expression levels (based on GFP fluorescence) by FACS. All cell lines in this study were maintained in Dulbecco’s Modified Eagle Medium (DMEM) supplemented with 10% fetal bovine serum (FBS) and 1X GlutaMax. All cells underwent monthly mycoplasma detection testing using the MycoAlert system (Lonza) [21, 37].

For data collection, cells were seeded at a density of 1–2 × 10^5^ cells/well in 6-well plates, 0.7–1.0 × 10^5^ cells/well in 12-well plates, or 0.5–0.7 × 10^5^ cells/well in 24-well plates and maintained for an additional 24 h before any treatment. Cells were treated with ISR stress inducers (300 nM thapsigargin (Sigma-Aldrich), 3 µM oligomycin (Sigma-Aldrich), 5 mM L-histidinol (Sigma-Aldrich), sodium arsenite (Sigma-Aldrich) or transfected with 250 ng/ml poly I:C (Tocris), as previously described [21], 1 µM ISRIB (Sigma-Aldrich), 50 µM Z-VAD-FMK (SelleckChem), or 1 µg/ml FcDR5 (R&D systems) as indicated. As previously described, FKBP-PKR was activated with 100 nM of the homodimerization ligand AP20187 (Takara) [21]. DR5 and eIF2α^S51D^ lentiviral plasmids were commercially synthesized and cloned into expression vectors using standard molecular biology techniques.

Stable cell lines bearing transgenes were generated by viral transduction as previously described [38]. Briefly, VSV-G pseudotyped retroviral particles encoding constructs of choice were prepared using standard protocols using GP2-293 packaging cells (Clontech). Viral supernatants were collected and used to infect target cells by centrifugal inoculation (spinoculation). For retroviral infections, target cells were plated at a density of 2 × 10^5^ cells/well in 6-well plates one day before transduction in presence of 2 µg/ml polybrene. Pseudoclonal cell populations were obtained by fluorescence-activated cell sorting using a narrow gate placed over the mean of the signal distribution as previously described [21].

### Immunoblotting

Cell lysates were collected directly in Laemmli SDS-PAGE sample buffer (62.5 mM Tris-HCl pH 6.8, 2% SDS, 10% glycerol, and 0.01% bromophenol blue). Lysates were briefly sonicated and supplemented with fresh 5% 2-mercaptoethanol before heat denaturation and separation by SDS-PAGE. Lysates were separated on 8–10% SDS-PAGE gels and transferred onto nitrocellulose membranes for immunoblotting. Immunoreactive bands were detected by enhanced chemiluminescence using horse radish peroxidase (HRP)-conjugated secondary antibodies. The antibodies and dilutions used were as follows: PKR (Cell Signaling Technology 3072, 1:2000), phospho-PKR (T466) (Abcam AB322036, 1:2000), eIF2α (Cell Signaling Technology 9722, 1:1000), phospho-eIF2α (Cell Signaling Technology 9721, 1:1000), FLAG (M2 clone Sigma-Aldrich F1804, 1:2000), ATF4 (Cell Signaling Technology 11815, 1:1000), CHOP (Cell Signaling Technology 2895, 1:1000), anti-DR5 (Cell Signaling Technology 8074, 1:1000), PARP1 (Cell Signaling 9532, 1:1000), caspase-8 (Cell Signaling Technology 9746, 1:1000), Caspase-3 (Cell Signaling Technology 9662, 1:1000) β-actin (1:5000, Sigma-Aldrich, 061M4808), anti-GAPDH (Abcam 8245, 1:5000), all diluted in 1% BSA-TBST. Secondary anti-rabbit and anti-mouse HRP-conjugated antibodies (Cell Signaling Technology 7074, 7076) were used at 1:5000 dilutions in 1% BSA-TBST. Images were acquired using a ChemiDoc imager (Biorad).

### Immunofluorescence analyses

H4 cells were grown on coverslips in 24-well plates, fixed with 4% PFA for 10 min, washed three times with PBS, and permeabilized with blocking solution (0.05% saponin, 0.5% BSA, 50 mM, NH_4_Cl in PBS) for 20 min. DR5 (Cell Signaling Technology 8074, 1:200), caspase-8 (Cell Signaling Technology 9746, 1:200), Flag (Sigma-Aldrich F1804, 1:200) and GM130 (BD technology 610822, 1:1000) primary antibodies were diluted in blocking solution and incubated for 1 h at room temperature. The coverslips were washed with PBS and incubated with fluorochrome-conjugated secondary antibodies (Alexa fluor anti-mouse 647; Invitrogen A32728 and Alexa fluor anti-rabbit 568; Invitrogen A11011 diluted at 1:500 dilution in blocking solution) and DAPI (0.1 µg/mL) for 45 min at RT. Fixed cells were washed 2 times in PBS and one time in ddH_2_O and mounted on coverslips with Mowiol. Images were acquired using a resonant scanning confocal microscope (Leica SP8) equipped with a Plan Apochromat 60 × NA 1.2 oil immersion objective. Fluorescence microscopy images were processed with Fiji (ImageJ: National Institutes of Health) software. To determine the proportion of DR5 in the *cis-*Golgi complex, each cell in a field of view was cropped out, and a single ROI was drawn manually to quantify the total DR5 fluorescence signal. GM130 signals were used to calculate the DR5 signal in the *cis-*Golgi complex using the Fiji plug-in “create selection.” An average of 200 cells per time point were collected for each replicate. The data were expressed as the ratio between DR5 MFI in the *cis*-Golgi compartment over the total DR5 MFI. Statistical significance for differences between groups was calculated using the unpaired Student’s t-test with the GraphPad Prism software. All data reported as mean ± s.e.m. Outliers were identified using the ROUT method in Graphpad Prism software.

### Cell viability assays

Cells were collected after trypsinization to measure viability by flow cytometry. Cells were resuspended in PBS supplemented with 2% FBS and 0.1 mg/ml RNase, and propidium iodide (PI, 1.5 µg/ml) was added to the cell suspension. The samples were incubated on ice for 10 min and separated in an Attune cytPix flow cytometer. Flow cytometry data was analyzed using FlowJo (TreeStar, USA). The proportions of live and dead cells determined by PI staining were used as cell viability metrics. Alternatively, cell viability was measured using Cell Titer Glo (Promega) following the manufacturer’s recommendations. Briefly, cells were grown at a density of 6 × 10^4^ cells/well in 96-well plates (40% confluence) and maintained for 24 h before any treatment. Different concentrations of ISR stressors were applied for an additional 24 h and viability readings (luminescence) were collected using a ClarioStar plate reader (BMG).

### Recombinant TRAIL treatments and ELISA

H4 FKBP-PKR cells were grown at a density of 6 × 10^4^ cells/well in 96-well plates and maintained for 24 h before any treatment. The homodimerization ligand AP20187 was added at a final concentration of 100 nM, and human recombinant TRAIL was added at different concentrations, as indicated in Figure S4A. Viability was measured using Cell Titer Glo (Promega) 24 h after treatment. Intracellular and extracellular TRAIL levels were analyzed with a human trail ELISA kit (R&D, DTLR00) according to the manufacturer’s instructions.

### DR5 neutralizing antibody assay

Cells expressing FKBP-PKR were washed twice with PBS and either fresh cell culture medium (control) or fresh cell culture medium supplemented with FcDR5 neutralizing antibody (1 µg/ml) were added, and the cells incubated overnight. The following day, the homodimerization ligand AP20187 was added to the cells and the cells were incubated for an additional 24 h before collection for analysis by immunoblotting or flow cytometry.

### qRT-PCR and RT-PCR analyses

Total RNA from sub-confluent H4 cells was isolated using the RNeasy RNA purification kit (Qiagen), following the manufacturer’s recommendations. 1 µg of total RNA was reverse transcribed with SuperScript VILO (Invitrogen) following the manufacturer’s recommendations. The resulting cDNA was used as a template for qRT-PCR using PowerUp SYBR Green Master Mix (Applied Biosystems), according to the manufacturer’s protocol. GAPDH, β-ACTIN, or 28S rRNA were used as normalizing controls to estimate fold changes in mRNA expression. Data were analyzed using GraphPad Prism, and outliers were identified using the ROUT method for *N* > 7. To measure XBP1 mRNA splicing, PCR was performed using Hot Start Taq DNA polymerase (NEB), and the PCR products were separated on 3% TAE-agarose gel. The oligonucleotide primers used in this study are provided in Table S1.

## Supplementary information

Supplementary material

Source Figures

## Data Availability

All data supporting this study are available in the article and supplemental material.
