# Supplementary material for: The integrated stress response engages a cell-autonomous, ligand-independent, DR5-driven apoptosis switch
Source: Cell Death Dis. 2025 Feb 15;16(1):101. doi: 10.1038/s41419-025-07403-8 (PMC11830069; doi:10.1038/s41419-025-07403-8)
Supplement: Supplementary file 1 — Supplementary material [file 41419_2025_7403_MOESM1_ESM.pdf]

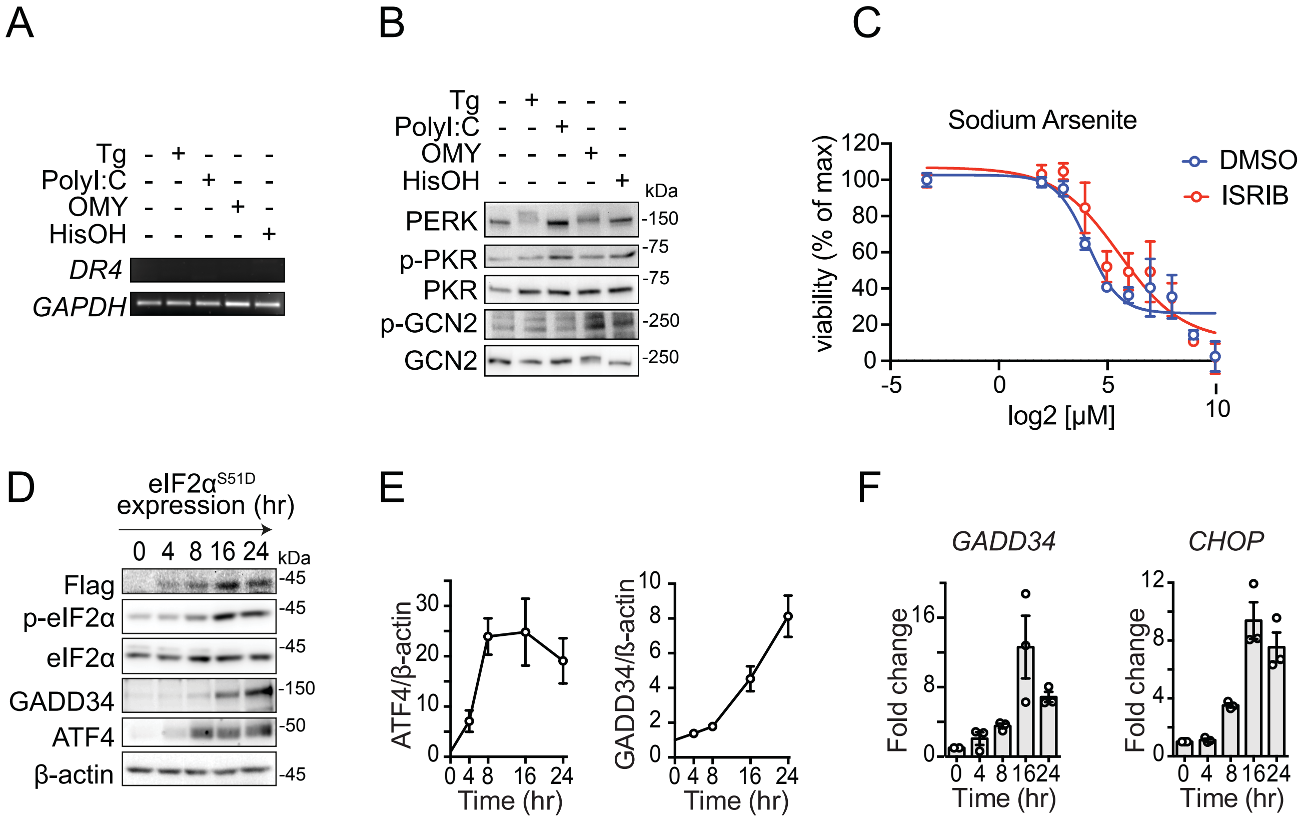
A
Tg - + - - -
PolyI:C - - + - -
OMY - - - + -
HisOH - - - - +
DR4
GAPDH
B
Tg - + - - -
PolyI:C - - + - -
OMY - - - + -
HisOH - - - - +
kDa
PERK -150
p-PKR -75
PKR -75
p-GCN2 -250
GCN2 -250
C
Sodium Arsenite
DMSO
ISRIB
viability (% of max)
log2 [μM]
D
eIF2α S51D expression (hr)
0 4 8 16 24
kDa
Flag -45
p-eIF2α -45
eIF2α -45
GADD34 -150
ATF4 -50
β-actin -45
E
ATF4/β-actin
Time (hr)
GADD34/ß-actin
Time (hr)
F
GADD34
CHOP
Fold change
Time (hr)

Figure S1

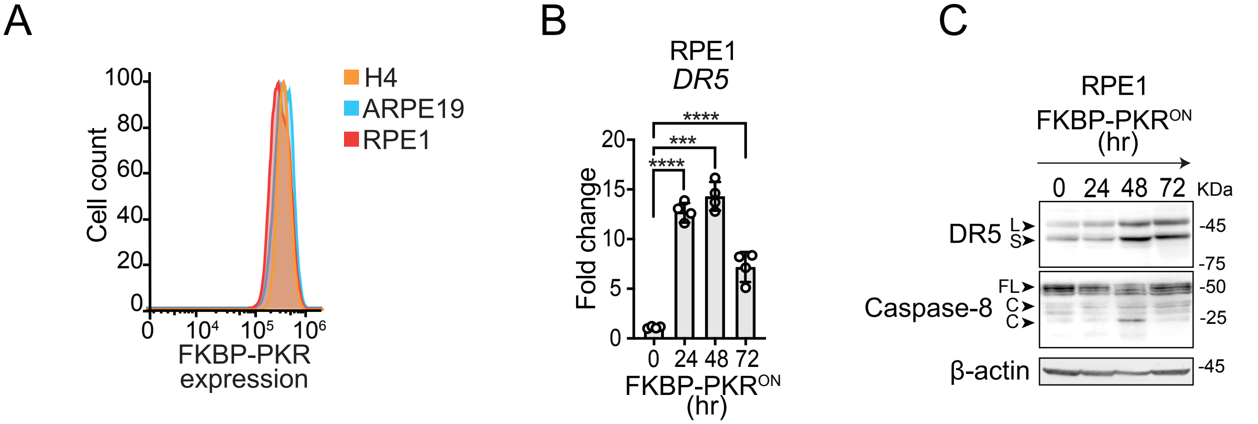

A
100
80
60
40
20
0
Cell count
0
10⁴
10⁵
10⁶
FKBP-PKR expression
H4
ARPE19
RPE1
B
RPE1
DR5
Fold change
20
15
10
5
0
****
***
****
0
24
48
72
FKBP-PKRON (hr)
C
RPE1
FKBP-PKRON (hr)
0
24
48
72
KDa
DR5
L
S
-45
-75
Caspase-8
FL
C
C
-50
-25
β-actin
-45


Figure S2

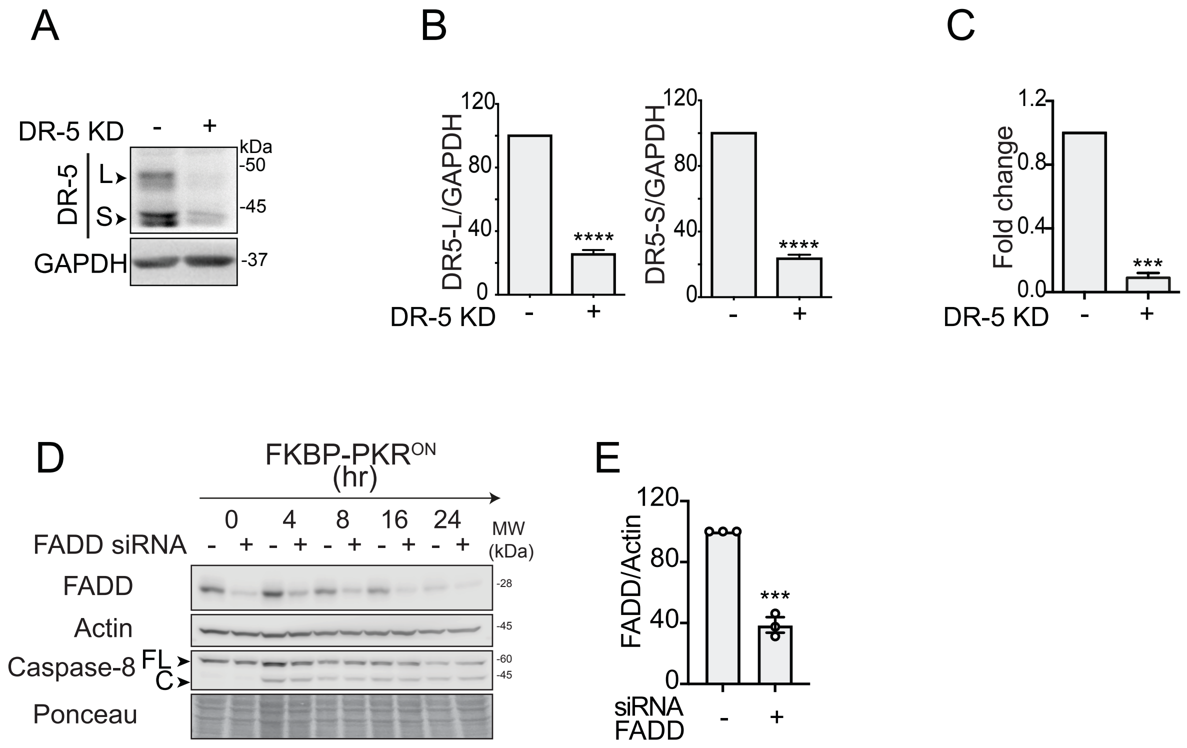
A
DR-5 KD - +
kDa
DR-5
L
S
-50
-45
GAPDH
-37
B
DR5-L/GAPDH
120
80
40
0
****
DR-5 KD - +
DR5-S/GAPDH
120
80
40
0
****
- +
C
Fold change
1.2
0.8
0.4
0.0
***
DR-5 KD - +
D
FKBP-PKR^ON
(hr)
0 4 8 16 24
FADD siRNA - + - + - + - + - +
MW
(kDa)
FADD
-28
Actin
-45
Caspase-8
FL
C
-60
-45
Ponceau
E
FADD/Actin
120
80
40
0
***
siRNA
FADD
- +

Figure S3

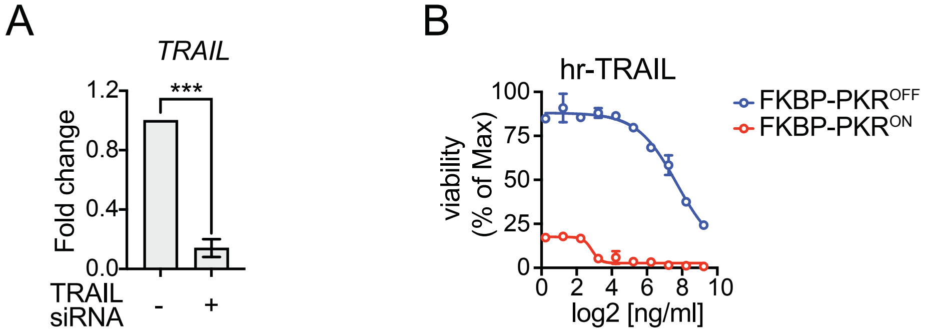
A
TRAIL
Fold change
***
TRAIL siRNA
-
+
B
hr-TRAIL
viability (% of Max)
log2 [ng/ml]
FKBP-PKR^OFF
FKBP-PKR^ON

Figure S4

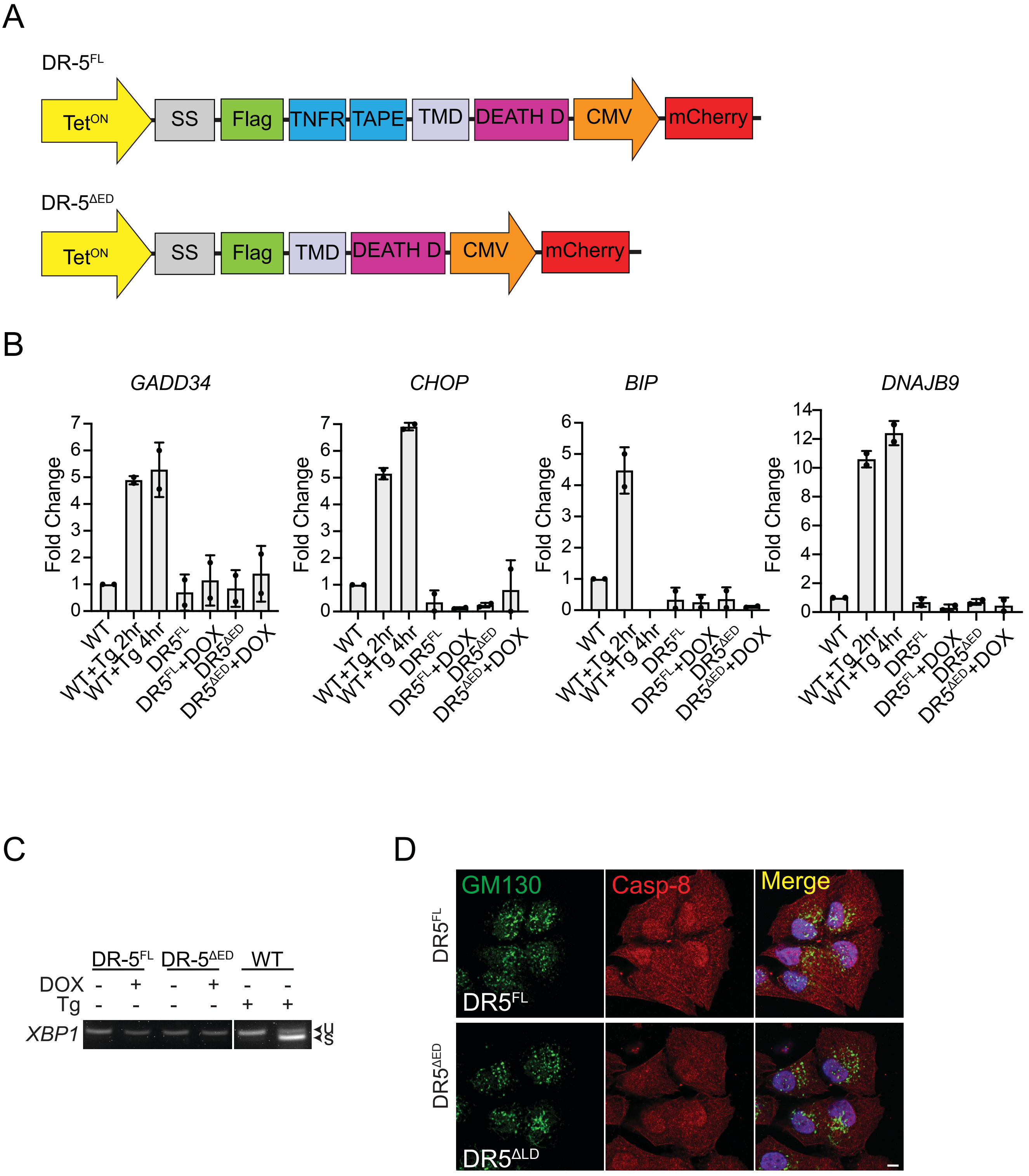

A
DR-5FL
TetON
SS
Flag
TNFR
TAPE
TMD
DEATH D
CMV
mCherry
DR-5ΔED
TetON
SS
Flag
TMD
DEATH D
CMV
mCherry
B
GADD34
CHOP
BIP
DNAJB9
Fold Change
WT
WT+Tg 2hr
WT+Tg 4hr
DR5FL
DR5FL+DOX
DR5ΔED
DR5ΔED+DOX
C
DR-5FL
DR-5ΔED
WT
DOX
Tg
XBP1
u
s
D
GM130
Casp-8
Merge
DR5FL
DR5ΔED
DR5FL
DR5ΔLD


Figure S5

**Table S1. RT-PCR oligonucleotide primers used in this study**

| Target | Primer sequence (Fwd) | Primer sequence (Rev) |
|---|---|---|
| DR5 | 5'-CCAGCAAATGAAGGTGATCC-3' | 5'-CGGTTTTGTTGACCCACTTT-3' |
| DR4 | 5'-TGAAGGGTCTCAGAGGAGGA-3' | 5'-CCATTTCATCAGCATTGCAT-3' |
| TRAIL | 5'-CACATAACTGGGACCAGAGGA-3' | 5'-CCTGAAATCGAAAGTATGTTTGG-3' |
| GADD34 | 5'-TGAGGCAGCCGGAGATAC-3' | 3'-GTAGCCTGATGGGGTGCTT-5' |
| CHOP | 5'-AGCCAAAATCAGAGCTGGAA-3' | 5'-CAGTGTCCCGAAGGAGAAAG-3' |
| β-ACTIN | 5'-TTCTACAATGAGCTGCGTGTG-3' | 5'-AGGGCATACCCCTCGTAGAT-3' |
| GAPDH | 5'-AGCCACATCGTCCAGACAC-3' | 5'-TGGAAGATGGTGATGGGATT-3' |
| XBP1 | 5'-GGAGTTAAGACAGCGCTTGG-3' | 5'-ACTGGGTCCAAGTTGTCCAG-3' |
| 28S | 5'-CTTACCAAAAGTGGCCCACTA-3' | 5'-AAACTCTGGTGGAGGTCCGT-3' |
| BIP | 5'-CTATTGGGGTGTTTCGCGAG-3' | 5'-GAGAGCTTCATCTTGCCAGC-3' |
| DNAJB9 | 5'-ACACTGGATCCAAGAAGCGT-3' | 5'-TTGAGTGACAGTCCTGCAGT-3' |

**Supplementary figure legends**

**Figure S1. Pharmacological and genetic ISR induction in H4 cells.** (A) Analysis of DR4 mRNA levels by RT-PCR in H4 cells after activation of the ISR with different pharmacological agents shows undetectable levels of DR4 transcript. GAPDH: loading control. Thapsigargin (Tg) 300 nM, poly I:C 250 ng/ml, oligomycin (OMY) 3 μM, L-histidinol (HisOH) 5 mM. (B) Western blot showing phosphorylation of the ISR sensor kinases in H4 cells upon treatment with pharmacological ISR inducers. (C) Viability curve of cells treated with different concentrations of sodium arsenite (SA) and co-treated with ISRIB (1μM). SA = 3.0 μM, $EC_{70}$ SA+ISRIB = 20.1 μM). (D) Western blot showing canonical ISR induction in H4 cells expressing FLAG epitope-tagged eIF2α$^{S51D}$ and treated with doxycycline for the indicated time. β-actin: loading control. (E) Densitometry quantification of the Western blot data in (C) for ATF4 and GADD34 (mean and SEM, N = 3). (F) qRT-PCR analysis of ATF4 and CHOP mRNA levels in H4 cells expressing FLAG epitope-tagged eIF2α$^{S51D}$ and treated with doxycycline for the indicated time (mean and SEM, N = 3).

**Figure S2. Synthetic ISR activation drives apoptosis in multiple cell types.** (A) Flow cytometry analysis of the levels of expression of FKBP-PKR in H4, ARPE19 and RPE1 cells using GFP expression as a proxy in cells transduced with a lentivirus encoding FKBP-PKR-P2A-GFP (see Materials and Methods for details). (B) qRT-PCR analysis of DR5 mRNA levels after activation of FKBP-PKR in RPE1 cells (mean and SEM, N = 4, ****$P$ < 0.0001, ***$P$ < 0.001, unpaired Student's t-test, non-parametric) (C) Western blot showing upregulation of DR5 isoforms and cleavage of caspase-8 after activation of FKBP-PKR in RPE1 cells. β-actin: loading control.

**Figure S3. Generation of DR5-depleted cell lines.** (A) Western blot showing the extent of CRISPRi-mediated knock-down of DR5 in H4 FKBP-PKR cells. GAPDH; loading control. (B) Densitometry quantification of the DR5 short and long isoforms upon genetic depletion by CRISPRi (mean and SEM, N = 3, ****$P$ < 0.0001, unpaired Student's t-test, non-parametric). (C) qRT-PCR analysis showing the levels of DR5 in H4 cells (mean and SEM, N = 3, ***$P$ < 0.001, unpaired Student's t-test, non-parametric). (D) Western blot showing the extent of RNAi-mediated knock-down of FADD in H4 FKBP-PKR cells and processing of caspase-8 at the indicated time points upon FKBP-PKR activation. Actin, Ponceau stain; loading controls. (E) Densitometry quantification of remaining FADD upon genetic depletion by RNAi (mean and SEM, N = 3, ***$P$ < 0.001, unpaired Student's t-test, non-parametric).

**Figure S4. TRAIL dependency for cell death upon ISR activation.** (A) Validation of TRAIL siRNA efficiency in H4 FKBP-PKR cells (mean and SEM, N = 3, ***$P$ < 0.001, unpaired Student's

t-test, non-parametric). (B) Quantification of cell viability of H4 FKBP-PKR cells co-treated for 24 hr with dimerizer and human recombinant TRAIL at the indicated concentrations (mean and SEM, N = 3).

**Figure S5. DR5 overexpression does not induce the UPR** (A) Schematic of DR5 constructs used in this study. (B) qRT-PCR analysis of UPR and ISR target genes in H4 $DR5^{FL}$ and $DR5^{\Delta ED}$ expressing cells. Tg: positive control. (C) RT-PCR showing no XBP1 mRNA splicing upon $DR5^{FL}$ and $DR5^{\Delta ED}$ expression. (D) Representative immunofluorescence images showing Golgi apparatus fragmentation and loss of caspase-8 localization to the Golgi apparatus upon brefeldin-A (0.1μg/ml for 16 hours) treatment in $DR5^{FL}$ and $DR5^{\Delta ED}$ expressing cells.
