# Supplementary material for: The integrated stress response engages a cell-autonomous, ligand-independent, DR5-driven apoptosis switch
Source: Cell Death Dis. 2025 Feb 15;16(1):101. doi: 10.1038/s41419-025-07403-8 (PMC11830069; doi:10.1038/s41419-025-07403-8)
Supplement: Supplementary file 2 — Source Figures [file 41419_2025_7403_MOESM2_ESM.pdf]

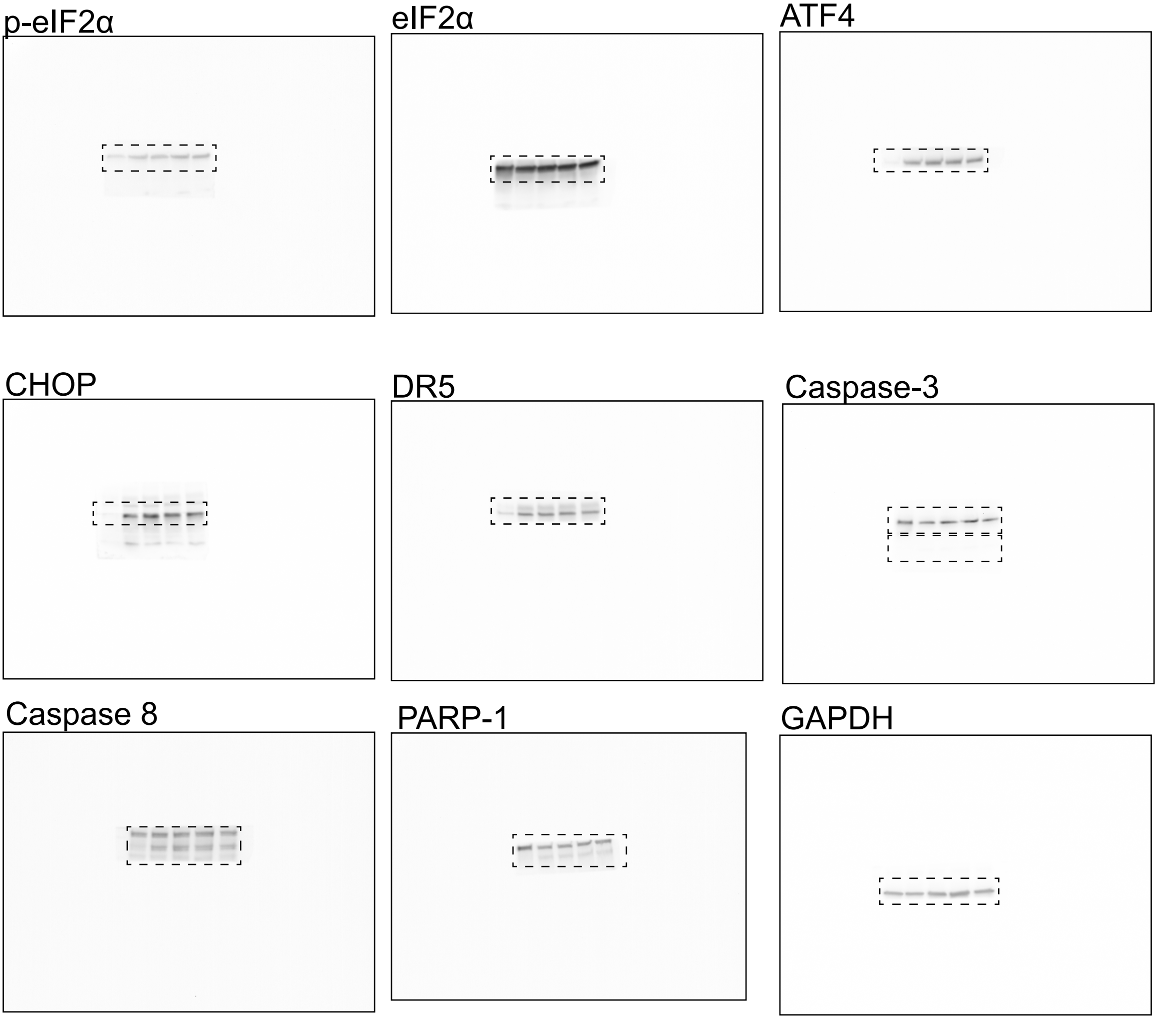

Source Figure 1B

DR5

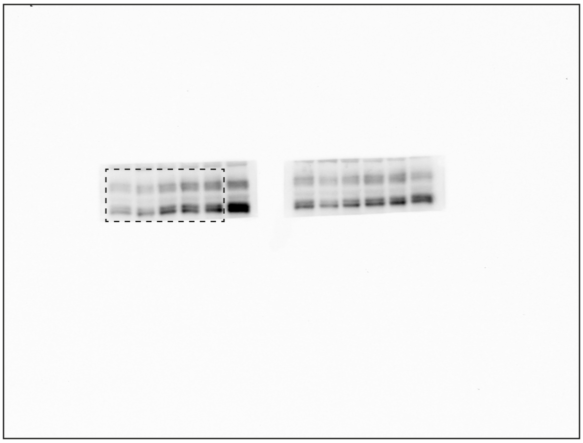

GAPDH

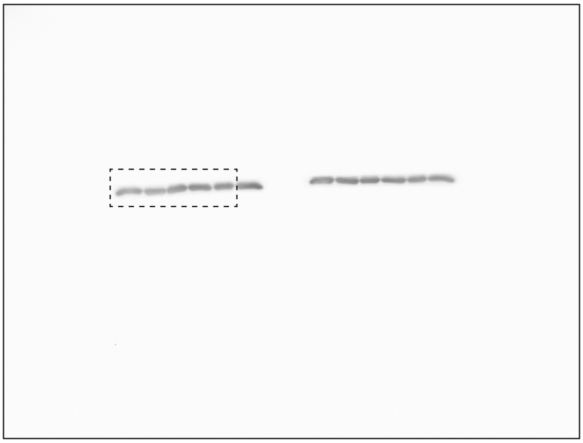

Source Figure 2B

CASP-8/ß-actin/ PARP1

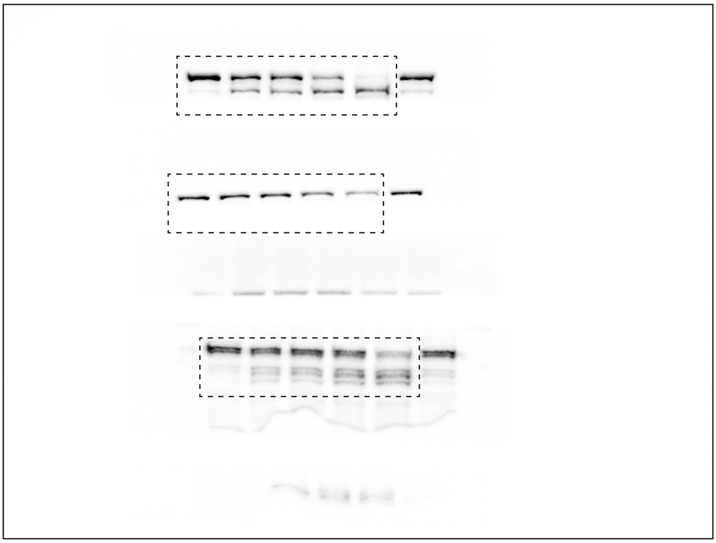

Source Figure 2D

Caspase-8 left_ARPE19_right_RPE1

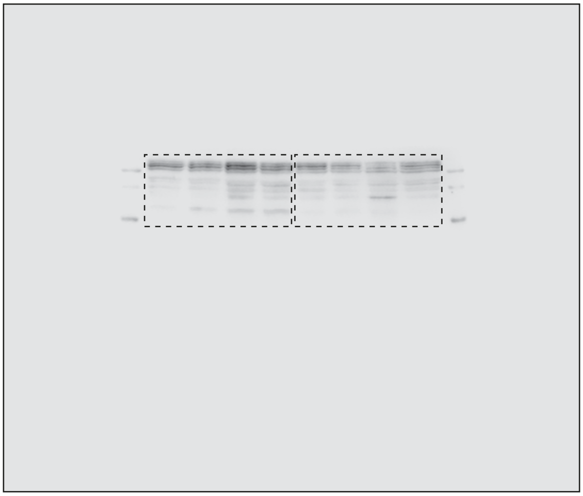

DR5_ARPE19

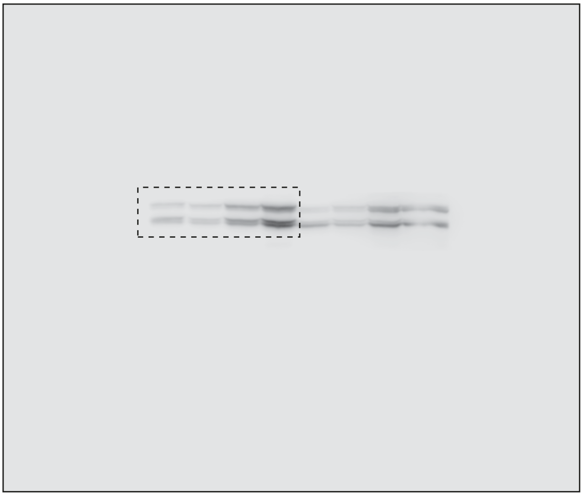

Actin_ARPE19

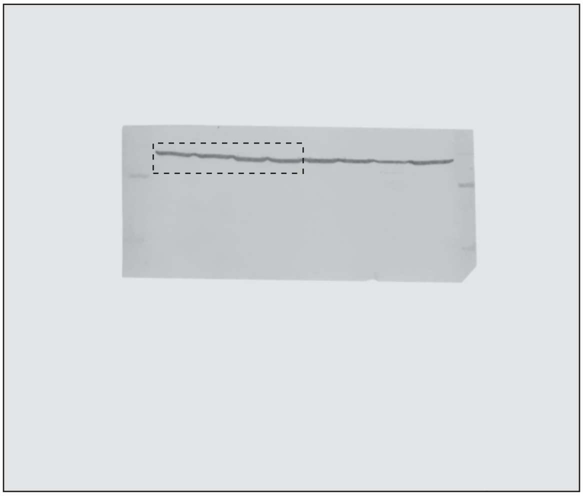

Source Figure 2F

Caspase-8

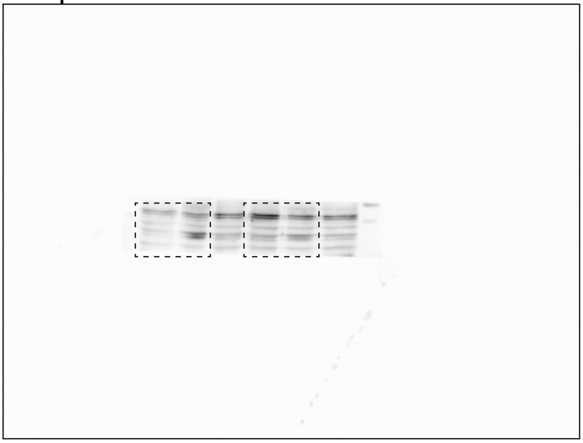

Active caspase-3

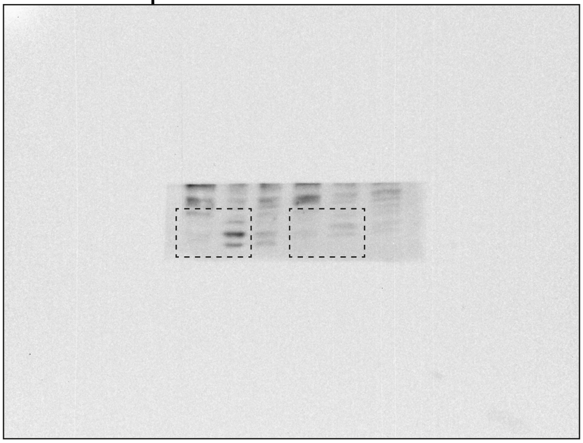

pro caspase-3

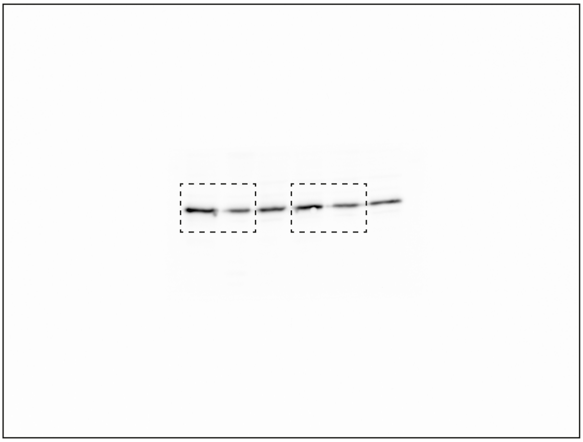

Source Figure 3A

PARP1

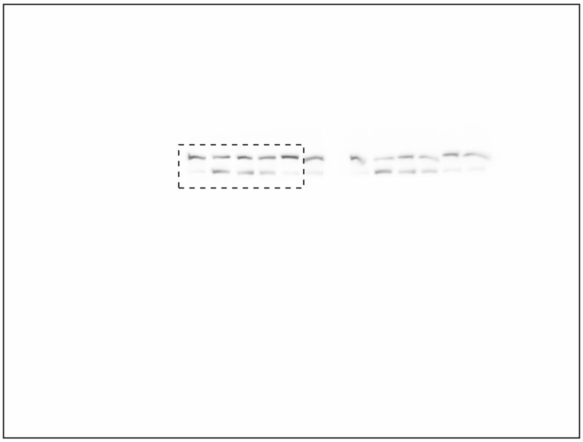

Pro-caspase 3

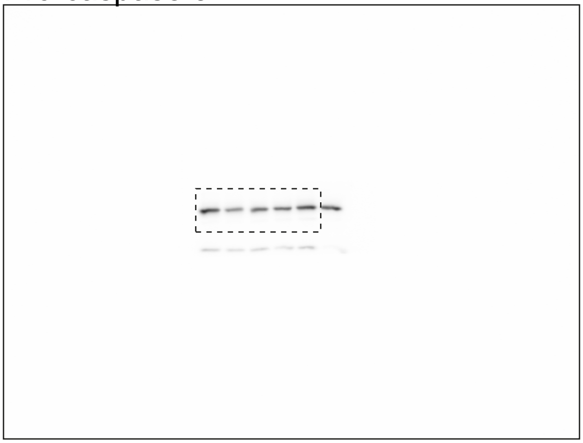

Active caspase 3

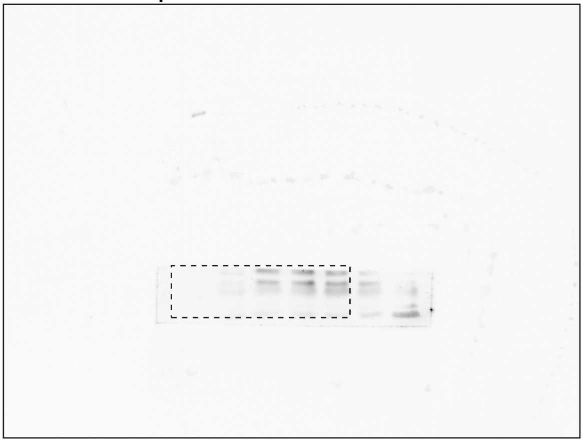

Caspase 8

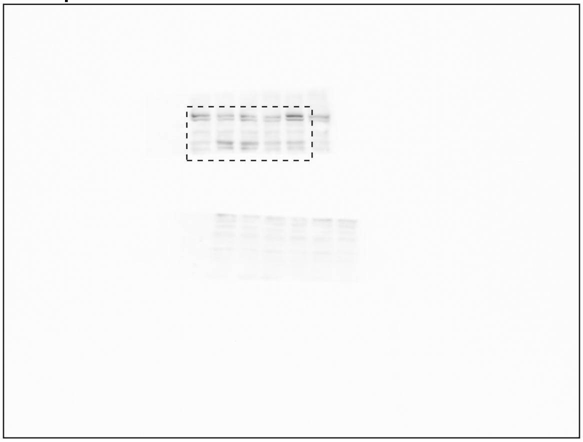

Source Figure 4D

DR5

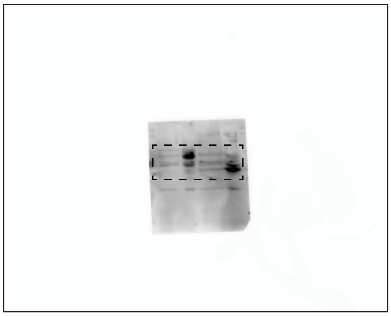

Ponceau of DR5

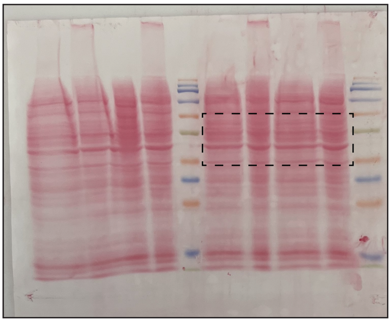

Flag

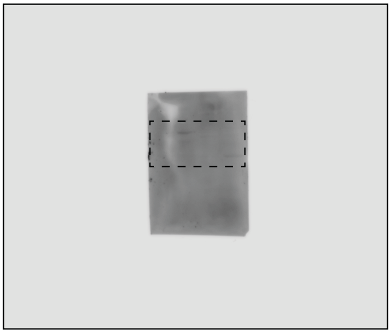

Ponceau of Flag

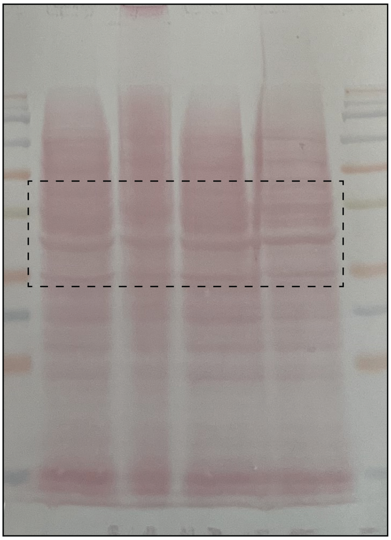

Source Figure 5C

Caspase 3

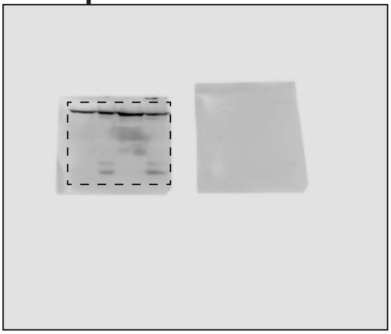

Caspase 8

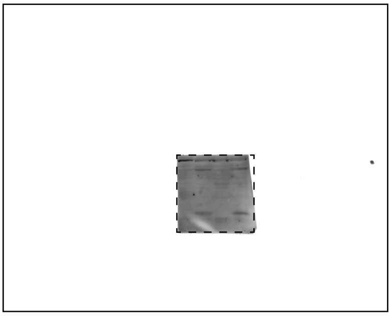

PARP1

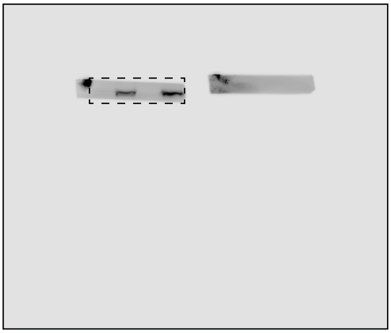

ß-Actin

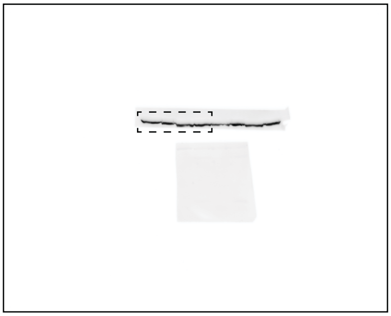

Source Figure 5E

DR4/ GAPDH

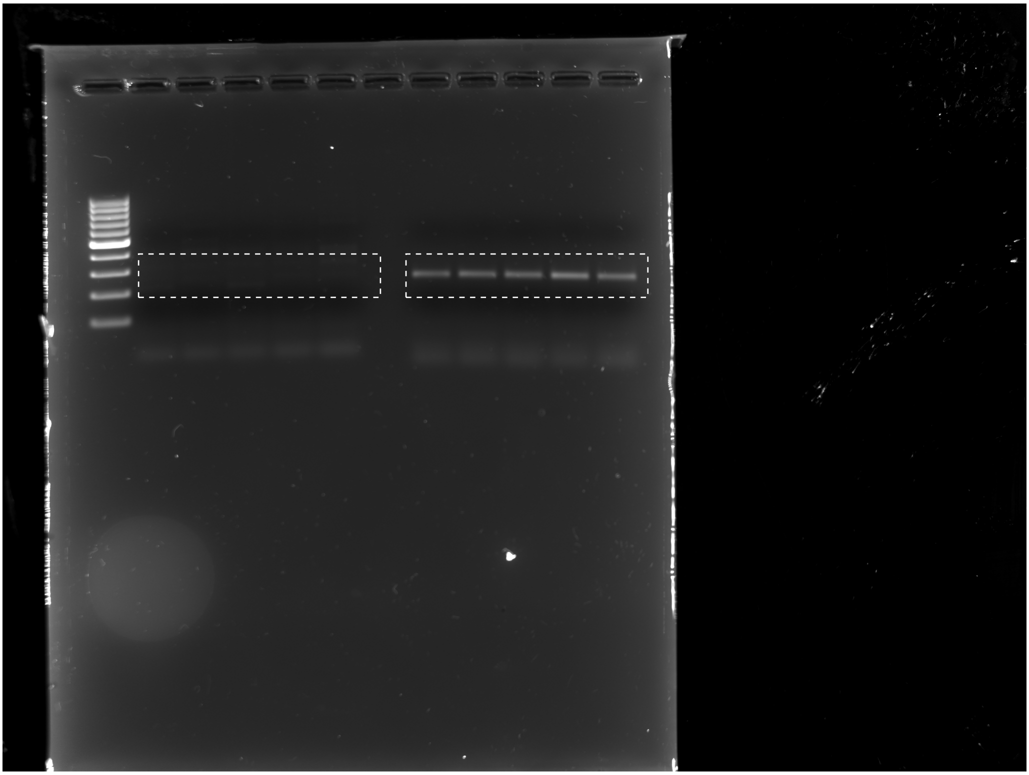

Source Figure S1A

p-GCN2

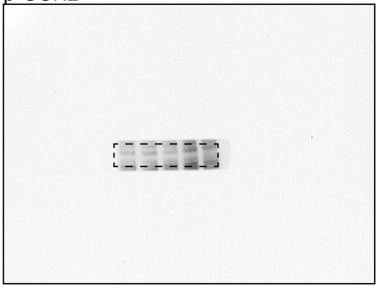

p-PKR

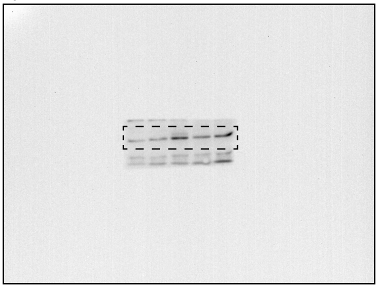

PKR

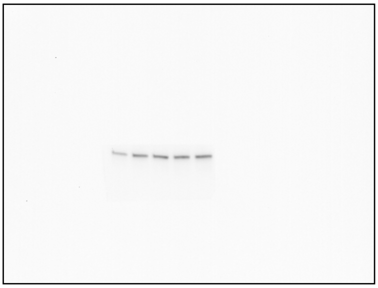

GCN2

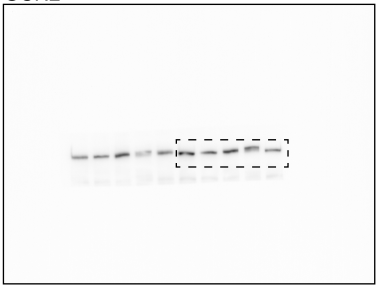

PERK

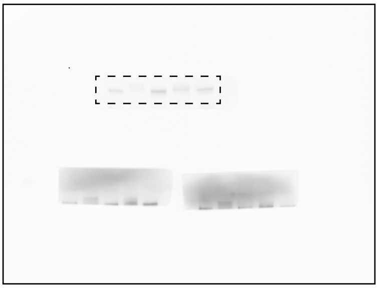

Source Figure S1B

- eIF2α

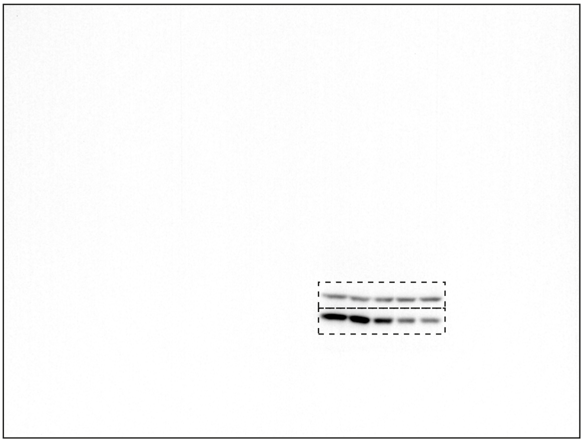

eIF2α

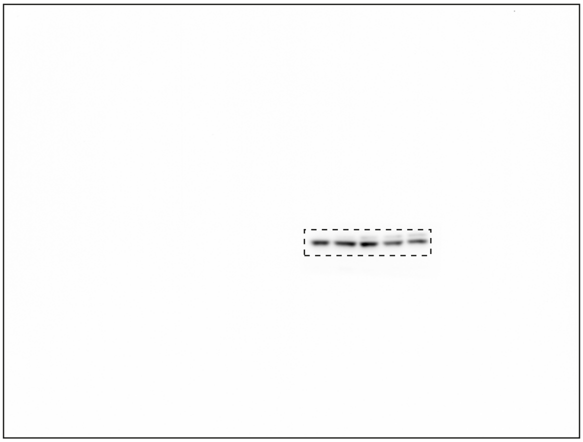

GADD34

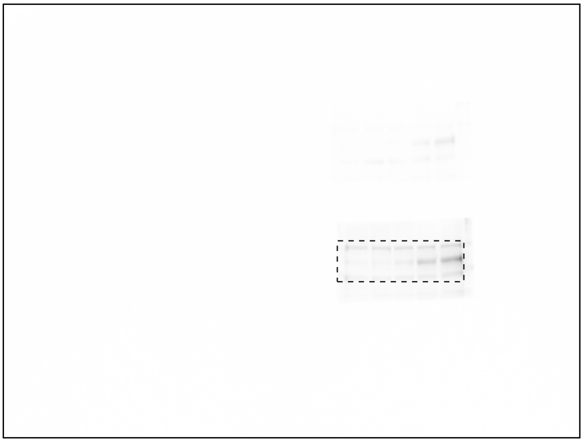

ATF4

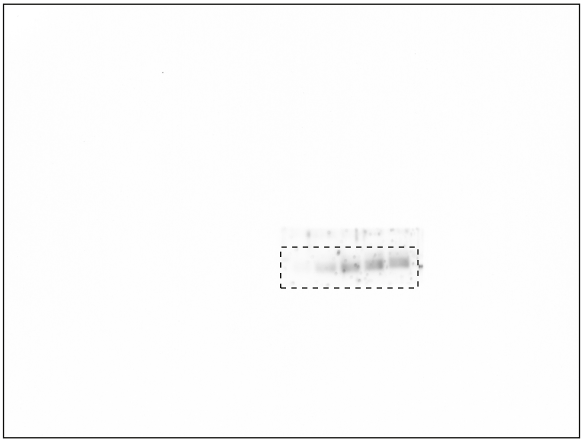

Flag

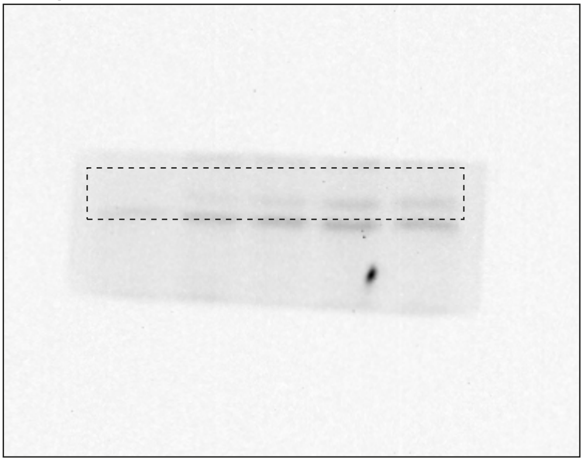

Source Figure S1D

Caspase-8 left_ARPE19_right_RPE1

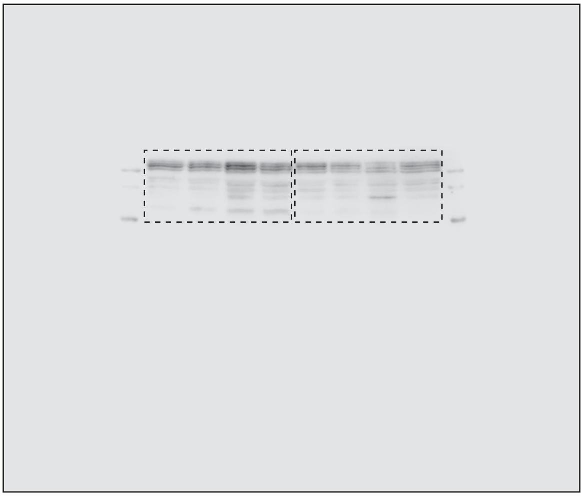

DR5_RPE1

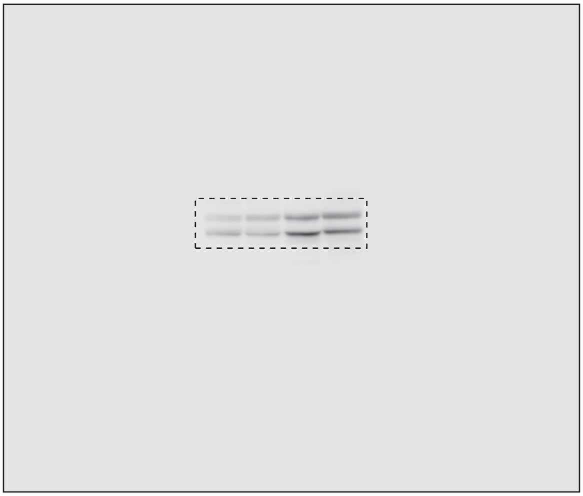

Actin_RPE1

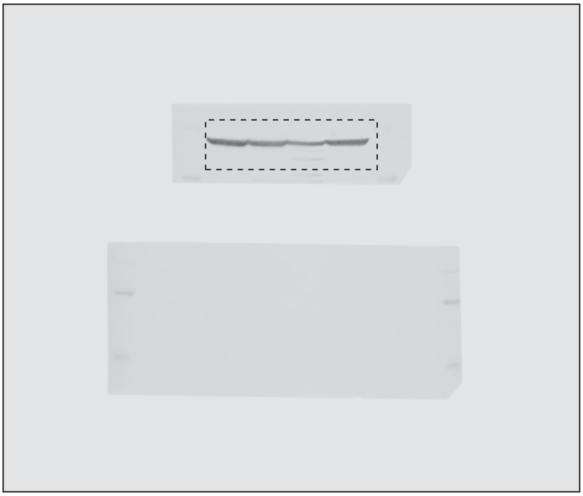

Source Figure S2C

DR5

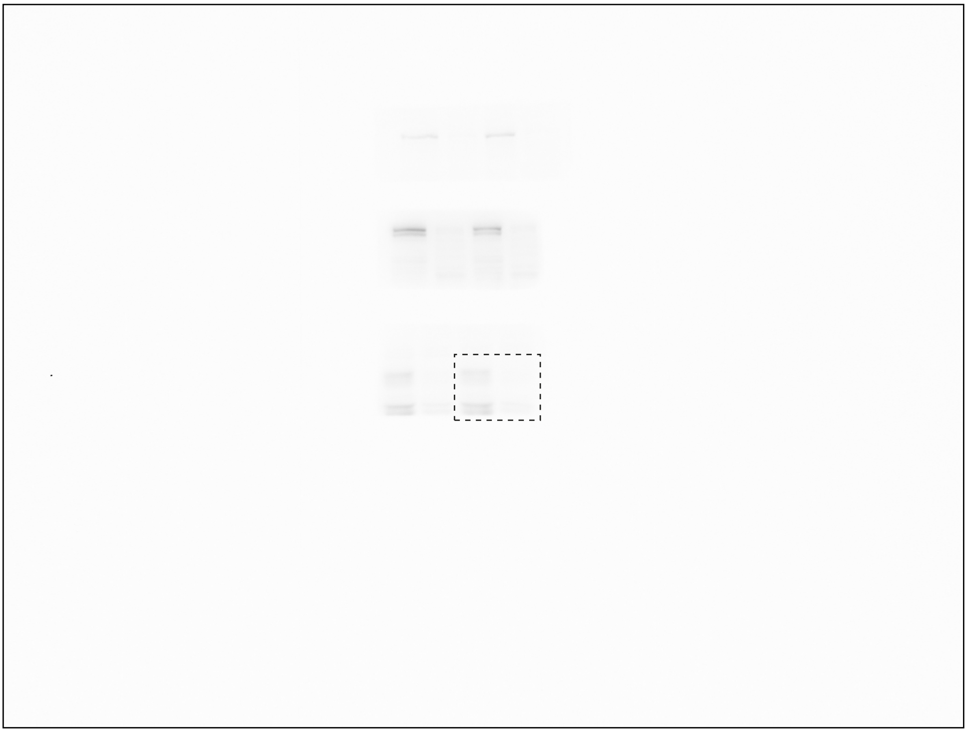

GAPDH

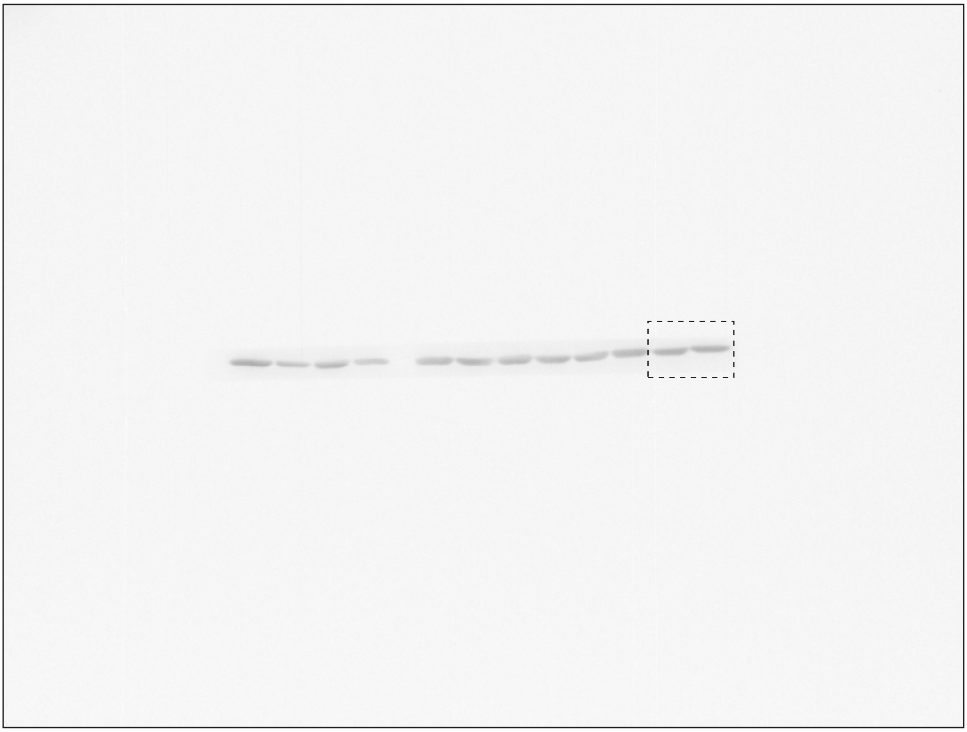

Source Figure S3A

XBP1

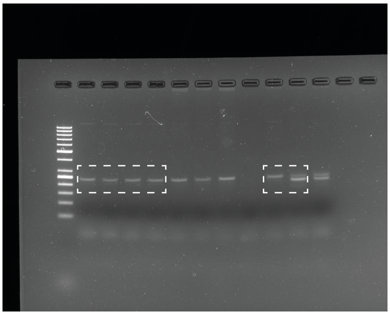

Source Figure S5C
